# Metabolic Plasticity in Ovarian Cancer Stem Cells

**DOI:** 10.3390/cancers12051267

**Published:** 2020-05-17

**Authors:** Alia Ghoneum, Daniela Gonzalez, Ammar Yasser Abdulfattah, Neveen Said

**Affiliations:** 1 Departments of Cancer Biology, Wake Forest University School of Medicine, Winston Salem, NC 27157, USA; aghoneum@wakehealth.edu (A.G.); gonzdm18@wfu.edu (D.G.); ammarabdulfattah@gmail.com (A.Y.A.); 2 Faculty of Medicine, University of Alexandria, Alexandria 21131, Egypt; 3 Departments of Pathology, Wake Forest University School of Medicine, Winston Salem, NC 27157, USA; 4 Departments of Urology, Wake Forest University School of Medicine, Winston Salem, NC 27157, USA; 5 Comprehensive Cancer Center, Wake Forest Baptist Health Sciences, Winston Salem, NC 27157, USA

**Keywords:** ovarian cancer, stem cells, metabolic plasticity, chemo-resistance

## Abstract

Ovarian Cancer is the fifth most common cancer in females and remains the most lethal gynecological malignancy as most patients are diagnosed at late stages of the disease. Despite initial responses to therapy, recurrence of chemo-resistant disease is common. The presence of residual cancer stem cells (CSCs) with the unique ability to adapt to several metabolic and signaling pathways represents a major challenge in developing novel targeted therapies. The objective of this study is to investigate the transcripts of putative ovarian cancer stem cell (OCSC) markers in correlation with transcripts of receptors, transporters, and enzymes of the energy generating metabolic pathways involved in high grade serous ovarian cancer (HGSOC). We conducted correlative analysis in data downloaded from The Cancer Genome Atlas (TCGA), studies of experimental OCSCs and their parental lines from Gene Expression Omnibus (GEO), and Cancer Cell Line Encyclopedia (CCLE). We found positive correlations between the transcripts of OCSC markers, specifically CD44, and glycolytic markers. TCGA datasets revealed that *NOTCH1, CD133, CD44, CD24,* and *ALDH1A1,* positively and significantly correlated with tricarboxylic acid cycle (TCA) enzymes. OVCAR3-OCSCs (cancer stem cells derived from a well-established epithelial ovarian cancer cell line) exhibited enrichment of the electron transport chain (ETC) mainly in complexes I, III, IV, and V, further supporting reliance on the oxidative phosphorylation (OXPHOS) phenotype. OVCAR3-OCSCs also exhibited significant increase in *CD36, ACACA*, *SCD*, and *CPT1A*, with *CD44, CD133,* and *ALDH1A1* exhibiting positive correlations with lipid metabolic enzymes. TCGA data show positive correlations between OCSC markers and glutamine metabolism enzymes, whereas in OCSC experimental models of *GSE64999, GSE28799*, and CCLE, the number of positive and negative correlations observed was significantly lower and was different between model systems. Appropriate integration and validation of data model systems with those in patients’ specimens is needed not only to bridge our knowledge gap of metabolic programing of OCSCs, but also in designing novel strategies to target the metabolic plasticity of dormant, resistant, and CSCs.

## 1. Introduction

Ovarian cancer (OvCa) is the fifth most common cancer in females and remains the most lethal gynecologic malignancy in the present day [1]. The five-year survival rate of women diagnosed with early localized disease is over 90%, but drops precipitously when diagnosed at stages III or IV [1]. The standard of care clinical management for OvCa includes debulking surgery followed by adjuvant chemotherapy or neoadjuvant chemotherapy followed by surgery [2]. Despite initial responses to chemotherapy, recurrence of chemo-resistant disease is encountered in almost 75% of patients with OvCa [2]. Recurrence has been attributed to suboptimal resection and the presence of residual chemo-resistant OCSCs [3,4] that hold the unique ability to adapt to environmental, metabolic, immunological, and pharmacological cues. While CSC clones may carry identical genetic signatures, increasing evidence has shown significant intra-clonal heterogeneity [5,6]. For example, some subsets of CSCs are maintained in a quiescent non-proliferative state in G0 phase, and upon environmental stimuli, may escape to reenter the cell cycle [7,8,9,10]. Of note, cellular quiescence is not a passive state, but rather is highly regulated by several pathways enabling CSCs swift reactivation [11,12,13]. The activated CSC subsets can undergo a phenotypic switch to a more proliferative epithelial-like state [14] accompanied by a metabolic shift towards increased aerobic glycolysis, OXPHOS, fatty acid oxidation (FAO), and glutaminolysis [15,16]. Active CSCs can metabolize glucose via the pentose phosphate pathway (PPP), producing an abundance of reduced NADPH and macromolecules that serve as the energetic building blocks needed for increased proliferation. Several factors affect this metabolic switch [17]. For example, hypoxic conditions favor a more undifferentiated CSC state with reduced proliferation and decreased cell-fate commitment [18]. Despite the fact that quiescent CSCs possess higher concentrations of ATP than their differentiated counterparts in solid tumors, they are less glycolytic [19,20]. Additionally, in glucose-deprived conditions, CSCs shift to a quiescent state and depend on OXPHOS for ATP production. Due to the extensive spatial and temporal heterogeneity of glucose, glutamine, and oxygen levels in the tumor itself, CSCs are forced to exhibit high metabolic plasticity to meet the increased demands of proliferation and metastasis [21]. Hence, OCSCs reprogram their metabolic and signaling machinery to maximize their survival and re-populate the tumor bulk [3]. This intrinsic ability of OCSCs to switch between different energy sources is viewed as “metabolic plasticity” and continues to pose as a challenge in cancer treatment [4]. Since OCSCs have a pluripotent undifferentiated phenotype, it is widely accepted that they exhibit metabolic plasticity and can switch between glycolysis, TCA cycle, FAO, glutaminolysis, and OXPHOS [22]. Importantly, oncogenes as Protein Kinase B (AKT), Hypoxia-Inducible Factor 1-alpha (HIF-1α), and tumor suppressors as p53 and Phosphatase and Tensin Homolog (PTEN) have been shown to play key roles in metabolic programing in OCSCs [23].

CSCs have been long established in hematologic malignancies; the first reports of CSCs in solid tumors were published in 2003 [24], demonstrating that breast CSCs exhibiting CD44^high^CD24^low^ cell surface markers isolated from metastatic tumors were able to self-renew and re-establish tumors when injected in immune-deficient mice [24]. In addition to CD44 and CD24, these CSCs expressed the transcriptional machinery associated with epithelial to mesenchymal transition (EMT), including Snail Family Transcriptional Repressor 1 (*SNAI1*/SNAIL), Snail Family Transcriptional Repressor 2 (*SNAI2*/SLUG), Twist-related protein 1 (*TWIST1*), and Zinc Finger E-Box Binding Homeobox 1 (ZEB1) concomitant with down-regulation of the epithelial marker E-cadherin and upregulation of mesenchymal markers, N-cadherin and vimentin [25]. Since these initial studies, CSCs have been reported to express stem cell surface markers as CD117/KIT as well as AC133, the epitope of CD133. [26]. Interestingly, several reports indicated that during differentiation, there is a pronounced decrease in the transcript and protein levels of AC133, but not of CD133 itself [27]. Transcription factors that are implicated in embryonal, hematopoietic, endothelial and neuronal stemness, and differentiation such as SRY (sex determining region Y)-box 2 (SOX2), POU domain, class 5, transcription factor 1/Octamer-binding transcription factor 4 (POU5F1/OCT4), Nanog homeobox (NANOG), and Notch homolog 1 (NOTCH1) have also been identified in CSCs [28]. Moreover, aldehyde dehydrogenase 1 enzyme (ALDH1), specifically its isotype *ALDH1A1*, has been recently identified as a useful CSC marker for further enrichment of subpopulations expressing one or more of the aforementioned stem cell markers [29]. It is noteworthy that the expression of CSC markers does not always correlate with disease stage or clinical outcome in many cancers due to their expression in small subpopulations or due to technical variations in measuring the expression of these markers [29].

OCSCs were first identified by Bapat and colleagues in 2005 in an experimental model system as a small subset of the cancer cells associated with sustained self-renewal, ability to drive tumor growth, metastatic dissemination, and chemo-resistance [30]. OCSCs have also been reported to express CSC markers that not only serve as markers of stemness, but were also implicated in different aspects of tumor growth, invasiveness, metastasis, chemo-resistance, and recurrence [31].

Most reports of OCSCs relied on *in vitro* phenotypic behavior, specifically their ability to form spheroids that express one or multiple CSC markers, and their resistance to the standard of care therapy. Chemo-resistant OvCa cells were also reported to exhibit the unique ability to form spheroids *in vitro* and express CSC markers as CD24, CD44, cKit/CD117, PROM1/CD133, ALDH1A1, SOX2, NANOG, POU5F1/OCT4, and NOTCH1 as well as multi-drug resistance markers [10,31,32,33,34,35,36,37]. Notably, the expression of markers of OCSCs does not depend on the OvCa subtype, but rather on environmental cues [38] as evidenced by varying expression of these markers in OCSC subpopulations under different *in vitro* cell culture conditions, along with the expression of distinctive transcriptomic signatures [38].

Several studies reported the signaling pathways implicated in the maintenance of cancer cells stemness. However, the metabolic pathways associated with the regulation of stemness are in infancy. CSCs were believed to exhibit the same metabolic programing as non-cancer stem cells, however, recent reports indicated that CSCs rely on multiple metabolic pathways depending on the cancer type, environmental cues, and the experimental model system that induces and/or supports the CSC phenotype [16,39,40]. The goal of this study is to unravel the correlations between putative stem cell markers with perturbed metabolic pathways in OvCa cells, OCSC model systems, as well as in patients’ tumors with the ultimate goal of bridging the knowledge gap in metabolic programing of OCSCs which can serve to guide researchers and physicians in developing and testing model systems and therapeutics targeting recurrent and resistant OvCa.

## 2. Material and Methods

### 2.1. Microarray Extraction

Gene expression profiles of two studies of OCSC *GSE28799* [41] and *GSE64999* [42] with platform information of GPL570 and GPL17077, respectively, were extracted from Gene Expression Omnibus (GEO). Both included ovarian cancer spheroids and their parental cells. *GSE28799* included OVCAR3-derived spheroids and their parental OVCAR3 in triplicates. *GSE64999* included undifferentiated spheroids and their parental differentiated spheroids in quadruplicates. Studies were selected using keywords: ovarian cancer and stem cells. Only data from studies with 3–4 biological replicates were used for analysis.

### 2.2. Data Analysis

The differential expression of the OCSCs markers and the enzymes involved in the metabolic pathways in OCSCs and their parental cells was analyzed by the multiple *t*-test with Holm–Sidak method, and each gene was analyzed individually between the two groups, without assuming a consistent SD with *p* ≤ 0.05. Data of the OvCa cell lines were downloaded from the Broad Institute Cancer Cell Line Encyclopedia (CCLE) portal (https://portals.broadinstitute.org/ccle/) and were similarly analyzed. The transcripts of OCSC markers and metabolic enzymes were correlated in the OCSC populations using Pearson’s correlation. All analyses were performed using GraphPad Prism 7.0 (San Diego, CA, USA). Correlations of the genes from The Cancer Genome Atlas (TCGA) data were performed using Gene Expression Profiling Interactive Analysis (GEPIA) web tool (http://gepia.cancer-pku.cn/) [43]. Bar graphs representing the prevalence of positive and negative correlations were generated in Microsoft Excel.

## 3. Results and Discussion

### 3.1. Correlation Between Putative OCSCs Markers

**Figure 1:**
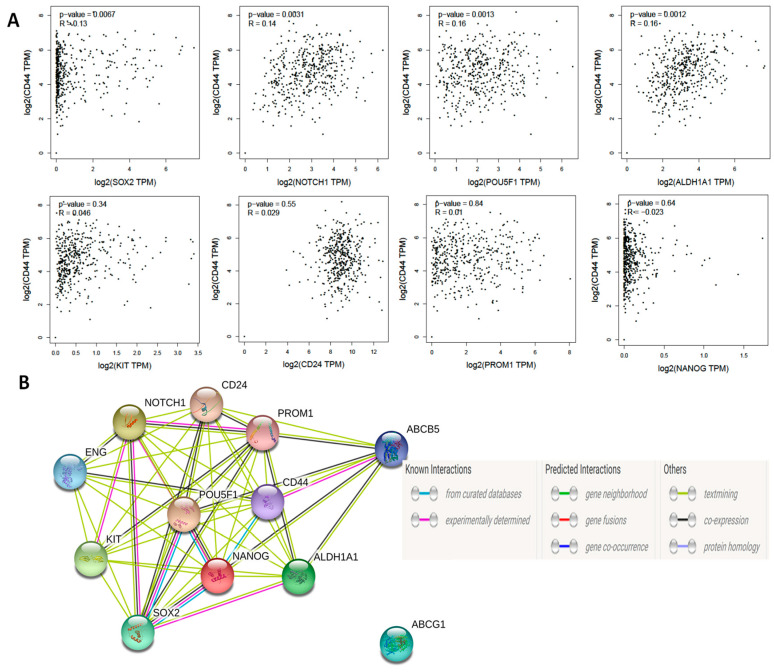
(**A**) Pearson’s correlation of CD44 with other ovarian cancer (OvCa) stem cell (OCSC) markers. (**B**) Predicted protein-protein interactions of OCSC markers using STRING web tool.

First, we sought to determine whether the various OCSC markers correlate with each other in patients’ tumors at the transcript level. Correlation analysis of TCGA data using GEPIA web tool revealed that *CD44* transcripts significantly correlated with other putative OCSCs markers *SOX2, NOTCH1, OCT4/POU5F1, ALDH1A1*, but not with *CD24, CD117/KIT, CD133/PROM1,* or *NANOG* (Figure 1A). It should be noted that CD44 contains over one hundred splice variants and is the most ubiquitous marker of CSCs. While not all CD44 variants have been correlated with cancer stemness, several prominent variants, including CD44v6 and CD44v8-10 were upregulated in a variety of epithelial malignancies, including ovarian cancer [44,45]. The presence of CD44v8-10 correlated strongly with transition of OCSCs to an epithelial phenotype in ascites while CD44v6 played a role in PI3K/AKT and MAPK pathways, and hence led to enhanced peritoneal dissemination [37,44,46,47]. Significant positive correlations were observed between *NOTCH1* and *CD133/PROM1* and *CD24,* between *CD117/KIT* and *ALDH1A1,* and between *SOX2* and *NANOG* (Figure S1). Interestingly, modeling the interactions of these OCSC markers using STRING protein-protein interaction web tool, predicted interactions based on experimental evidence between NOTCH1, SOX2, OCT4, NANOG, CD117, and CD133; whereas CD44 connected with drug resistance marker ABCB5 (Figure 1B).

**Figure 2:**
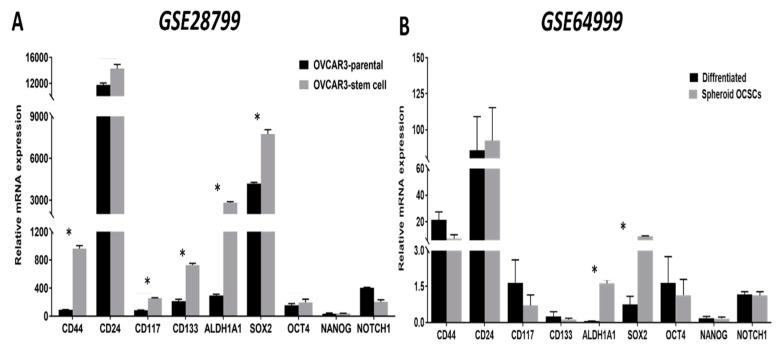
Expression of OCSC markers in OvCa stem cells and their parental cells. Bars represent mean ± SEM of the relative mRNA expression of the indicated OCSC markers in (**A**) OVCAR3 stem-cells vs. their parental cells (*n* = 3 each) in *GSE28799*, and (**B**) differentiated vs. undifferentiated OCSCs (*n* = 4 each) in *GSE64999.* Significance was determined using multiple *t-*test and Holm–Sidak method with each row analyzed individually with *p* < 0.05, without assuming a consistent SD.

Next, we analyzed data of publicly available datasets from studies that compared OCSCs with their parental cells. In a study that characterized OCSCs from OVCAR3 cell line, *GSE28799* [41], we found that, consistent with the original report, *CD44, CD24,* CD117*/KIT,* CD133/*PROM1, ALDH1A1,* and *SOX2* were significantly upregulated in OVCAR3 spheroid- derived stem cells compared to their parental OVCAR3 cells. No significant changes were detected in *OCT4/POU5F1* and *NANOG*, however, *NOTCH1* expression was significantly down-regulated in spheroid-derived OCSCs compared to parental OVCAR3 cells (Figure 2A). In another study *GSE64999* [42], CSC-related features of established serially diluted spheroid cells were examined *in vitro* under serum-containing and CSC culture conditions (will be referred to thereafter as differentiated and undifferentiated OCSCs, respectively). In this study [42], differentiated spheroids cultured in the presence of serum-containing media underwent epithelial differentiation with epithelial-like morphology and reduction of stem cell markers *ALDH1A1* and *SOX2* compared to parental undifferentiated spheroids cultured in stem cell media. Further analysis of the associated *GSE64999* dataset confirmed the significant upregulation of *ALDH1A1* and *SOX2* but not the other putative OCSCs’ markers (Figure 2B).

### 3.2. Correlation between OCSCs Markers and Glycolysis

Glycolysis is an oxygen-independent metabolic pathway that occurs in the cytosol, generating ATP from the conversion of glucose into pyruvate. Glycolytic metabolic reprogramming is critical for the maintenance of CSCs and is associated with cancer progression and chemo-resistance [48]. OCSCs share a similar pattern of glycolytic events with CSCs in cancers of the brain, breast, lung, liver, and bone, that they significantly increase their glucose uptake and lactate production compared with their non-CSC counterparts [49,50,51].

**Figure 3:**
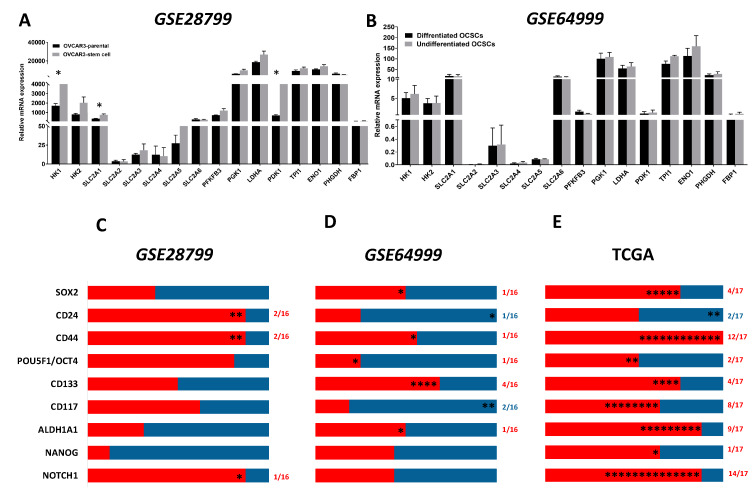
Expression of glycolysis markers in *GSE28799* and *GSE64999: (***A**,**B**) Bars represent mean ± SEM of the relative mRNA expression of the indicated glycolytic enzymes in OVCAR3 stem-cells vs. their parental cells in *GSE28799,* and undifferentiated vs. differentiated OCSCs in *GSE64999*, respectively. Significance was determined using multiple *t*-test and Holm–Sidak method with each row analyzed individually with *p* < 0.05, without assuming a consistent SD. (**C**–**E**) Bar graphs represent the prevalence of positive (**red**) and negative (**blue**) correlations of the indicated markers in OVCAR3-spheroids and the glycolytic enzymes in Supplement Tables S1–S2 and Table 1. * indicates the number of significant correlations either positive or negative.

We first compared the expression of the enzymes involved in glycolysis in OCSCs and their parental cells in both *GSE28799* and *GSE64999*. We found that the glucose transporter *SLC2A1, Hexokinase 1 (HK1),* and pyruvate dehydrogenase kinase 1 (*PDK1*) were significantly upregulated in OVCAR3 spheroid-derived OCSCs compared to their parental OVCAR3 cells (Figure 3A). In contrast, there was no significant difference in glycolytic enzymes between undifferentiated and differentiated spheroids in *GSE64999* (Figure 3B). There was a general trend of positive correlations between OCSC markers and glycolytic enzymes in OVCAR3-spheroids OCSCs compared to their parental OVCAR3. *CD44* and *CD24* exhibited significant positive correlation with *HK1* and phosphoglycerate kinase 1 (*PGK1*). *NOTCH1* exhibited significant positive correlation with *HK2.* None of the other stemness markers exhibited significant positive correlation with glucose transporters or glycolysis (Figure 3C and Table S1). Consistently, OCSC markers in undifferentiated spheroids in *GSE64999*, did not exhibit a glycolytic enrichment. *CD44* positively correlated with *SLC2A6,* while *CD24* negatively correlated with *PHGDH*. *CD117/KIT* exhibited significant negative correlations with *SLC2A1* and *SLC2A5. CD133/PROM1* exhibited significant positive correlation with *HK2, PGK1, PDK1*, and enolase1 (*ENO1*). *ALDH1A1* and *SOX2* positively correlated with *SLC2A3*, whereas *POU5F1/OCT4* and *NANOG* positively correlated with *SLC2A2*. However, *NOTCH1* did not exhibit any significant correlation with glycolytic enzymes or glycolysis (Figure 3D and Table S2).

Analysis of patients’ tumors, from TCGA data revealed that *CD44* and *NOTCH1* were the top CSC markers whose expression exhibited positive correlations with glucose transporters and glycolytic enzymes (Figure 3E and Table 1). *NOTCH1* positively correlated with six glucose transporters (*SLC2A1-6*), *HK1* and *HK2*, the rate limiting enzymes in glycolysis, and nine out of thirteen mapped enzymes involved in glycolysis. Similarly, *CD44* was significantly positively correlated with three out of the six glucose transporters, hexokinases as well as nine out of thirteen enzymes involved in glycolysis. *CD117/KIT* was positively correlated with *SLC2A1-4*, *HK2*, and two out of thirteen mapped glycolytic enzymes, 6-phosphofructo-2-kinase/fructose-2,6-biphosphatase 3 (*PFKFB3)* and phosphoglycerate dehydrogenase (*PHGDH)*; *CD133/PROM1* correlated with *HK1* and *HK2*, and two out of the thirteen enzymes in glycolysis. ALDH1A1 correlated with *HK1*, *SLC2A1,* and seven out of the thirteen enzymes involved in glycolysis. *SOX2* correlated positively with *SLC2A3* and four out of the thirteen enzymes in glycolysis. Other cell stemness markers as *CD24, OCT4,* and *NANOG* did not significantly correlate with glucose transporters, but positively correlated with only two of the glycolytic enzymes.

Transcriptomic profiling of data of the OvCa cell lines in CCLE revealed that the transcripts of *CD44* positively correlated with a glycolytic signature with significant positive correlation with *HK2,* lactate dehydrogenase A (*LDHA),* and *ENO*1, but a negative correlation with *SLC2A4* (Figure S2). *CD117/KIT* exhibited a positive correlation with *SLC2A4* and fructose-bisphosphatase (*FBP1)* which catalyzes hydrolysis of fructose 1,6-bisphosphate to fructose 6-phosphate, the rate-limiting step in gluconeogenesis. *CD133/PROM1* expression significantly negatively correlated with *SLC2A3, PFKFB3, LDHA*, and triosephosphate isomerase 1 (*TPI1),* and positively correlated with *FBP1* and *PHGDH*. *ALDH1A1* negatively correlated with *SLC2A4* and *SLC2A6*. *SOX2* expression is negatively correlated with *PFKFB3* and positively correlated with *SLC2A2* and *SLC2A4*. *POUF5F1/OCT4* was negatively correlated with *HK1, SLC2A3,* and *PHGDH* and positively correlated with *SLC2A1*. *NANOG* negatively correlated with *PGK1, LDHA, TPI1,* and positively correlated with *PHGDH. NOTCH1* was negatively correlated with *SLC2A5* and positively correlated with *LDHA* (Figure S2).

Taken together, data from *GSE28799* and TCGA show positive correlations between the transcripts of OCSC markers and glycolytic markers, whereas in *GSE64999,* fewer positive correlations were observed with a tendency towards a negative correlation with glycolytic markers. These discrepancies may be due to technical variabilities in experimental model systems using different cell lines under diverse cell growth media; whereas in patients’ tumor samples, the correlations are influenced by tumor heterogeneity and environmental cues. The high glucose and supplemental growth factors and serum present in culture media, combined with normoxic conditions and other pharmacologic inhibitors as Rho-associated protein kinase (ROCK) inhibitor used in the OCSC model in *GSE64999* [42] do not represent the glucose and oxygen deprived conditions resulting from dysfunctional vasculature and lack of perfusion that characterizes the OvCa tumor microenvironment (TME) [52]. Specifically, OvCa cells are highly sensitive to oxygen conditions; it is known that to survive hypoxic conditions, OCSCs are forced to upregulate their stem-like properties and behave more aggressively when brought back to higher oxygen environments [53]. Hypoxia also induced a decrease in OXPHOS and fatty-acid desaturation [52]. Further limitations that should be considered are that the *in vitro* culture models lack vital extracellular matrix components including immune cells, stromal cells, and structural matrices, all of which are known to influence OCSC expression, growth, and differentiation. The absence of biophysical properties including interstitial flow, oxygen partial pressure, and surrounding environmental stiffness as well as the lack of biochemical cues are all factors that must be considered when comparing cell culture systems to *in vivo* CSC properties [54]. In CCLE databases, the correlations represent the behavior of OvCa cell lines under optimal growth conditions. The expression of *CD44* positively and significantly correlated with most glycolytic phenotype in OCSC model systems, patients’ tumors as well as non-stem OvCa cells. This finding is consistent with earlier reports that CD44 is crucial for the regulation of glycolytic metabolism [55].

### 3.3. Correlation between OCSCs Markers and TCA Cycle

**Figure 4:**
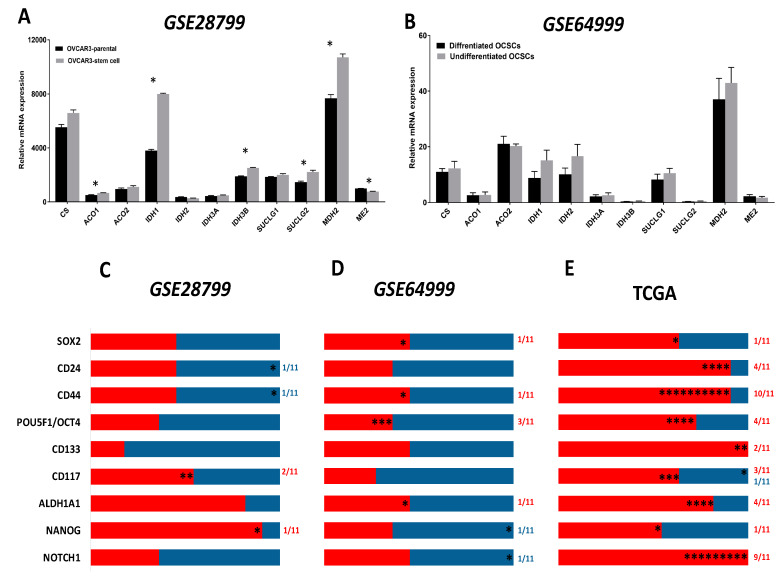
Expression of TCA markers in OCSC models. (**A**,**B**) Bars represent mean ± SEM of the relative mRNA expression of the indicated TCA enzymes in OVCAR3 stem-cells vs. their parental cells in *GSE28799* and undifferentiated vs. differentiated OCSCs in *GSE64999.* Significance was determined using the multiple *t*-test and Holm–Sidak method with each row analyzed individually with *p* < 0.05, without assuming a consistent SD. (**C**–**E**) Bar graphs represent the prevalence of positive (**red**) and negative (**blue**) correlations of the indicated OCSC markers and TCA enzymes in Supplement Tables S3–S4 and Table 2. * indicate the number of significant correlations either positive or negative.

The TCA cycle is a hub for the integration of multiple catabolic and anabolic pathways, as glycolysis, gluconeogenesis, mitochondrial electron transport chain, fatty acid, and cholesterol synthesis as well as glutamine metabolism. TCA cycle generates metabolic intermediates that are not only critical for anabolic and catabolic pathways and redox homeostasis, but are also implicated in the regulation of transformation, carcinogenesis, inflammation, and immunity [22,56,57,58]. The expression and function of the TCA enzymes and metabolites in cancer cells in general and OvCa in particular are unfolding; however, in OCSCs, these are not yet unraveled. Therefore, we sought to determine the association of TCA enzymes with OCSC model systems and in correlation with OCSC markers in patients’ specimens and model systems. We further analyzed the expression of the transcripts of TCA cycle enzymes in OVCAR3-stem cells and their parental controls in *GSE28799*. We found that aconitase 1 (*ACO1*), isocitrate dehydrogenase (*IDH1), IDH3*A, succinate-CoA ligase GDP-forming subunit beta (*SUCLG2*), and malate dehydrogenase 2 (*MDH2*) were significantly upregulated in OVCAR3-stem cells, whereas malic enzyme 2 (*ME2*) was significantly downregulated (Figure 4A). In *GSE64999,* no significant differences were found between the transcripts of TCA cycle enzymes between differentiated and undifferentiated OCSCs (Figure 4B). In *GSE28799*, there was a correlation between the expression of the enzyme transcripts and OCSC markers in OVCAR3-stem cells revealed a trend towards positive correlations with TCA enzymes. NANOG positively correlated with ten enzymes with significance only with *CS. ALDH1A1* exhibited positive though insignificant correlations with nine enzymes. *CD117/KIT* positively correlated with six enzymes with significance only with *IDH2* and *MDH2.* Other factors exhibited positive though insignificant correlations with TCA enzymes. *CD44* and *CD24* exhibited similar five positive and six negative correlation patterns with significant negative correlation with *ME2*. However, significant negative correlations were observed between CD133 with *IDH3B,* and *SOX2* with *IDH2.* Neither *POU5F1*/*OCT4* nor *NOTCH1* bear significant correlation with key TCA enzymes (Figure 4C and Table S3). Correlating the expression of CSC transcripts with those transcripts of TCA in undifferentiated and differentiated spheroids in *GSE64999* revealed a more negative trend. *CD44* positively correlated with five enzymes with significance only with *ACO1*. *ALDH1A1* and *SOX2* exhibited identical five positive and six negative profiles with significant positive correlation with *SUCLG1*. *POU5F1/OCT4* positively correlated with four enzymes, of them significance was noted with *citrate synthase* (*CS), IDH3B,* and *SUCLG1*. *NANOG* negatively correlated with seven enzymes with significant negative correlation with *MDH2;* while NOTCH1 negatively correlated with six enzymes with significance with *ACO1*. CD24, CD117, and CD133 were all not found to be significantly correlated with enzymes involved in the TCA cycle (Figure 4D and Table S4).

Analysis and correlation of the OCSCs’ markers with TCA enzymes from TCGA datasets (Figure 4E and Table 2) revealed that *NOTCH1* positively correlated with all TCA enzymes with significant correlations with all enzymes except *SUCLG1/2.* Similarly, *CD133* positively correlated with all eleven TCA enzymes with significance observed with *CS* and *ACO1*. *CD44* positively and significantly correlated with ten out of the eleven enzymes namely *CS, ACO1/2, IDH1/2/3A, SUCLG1/2, MDH2*, and *ME2*, while *CD24* positively correlated with ten out of the eleven enzymes with significant correlations with *CS, ACO2, CD117, SUCLG1,* and *ME2*. *ALDH1A1* positively correlated with nine out of the eleven enzymes with significance observed with *CS, ACO1, IDH1*, and *SUCLG1*. *POU5F1/OCT4* positively correlated with eight out of the eleven enzymes with significance observed with *ACO1/2, SUCLG2,* and *ME2*. *CD117*/*KIT* positively correlated with seven out of eleven enzymes with positive significance observed with *CS, ACO1,* and *IDH2* and significant negative correlation with *MDH2*. *SOX2* positively correlated with seven enzymes with significance observed only with *ACO1,* whereas *NANOG* positively correlated with six enzymes with significance observed with *CS.*

Correlation of OvCa cell line data acquired from CCLE demonstrated a generalized trend of negative correlation between OCSC markers and TCA cycle enzymes. Although *CD117/KIT* positively correlated with all enzymes, no significance was noted. *SOX2* positively correlated with eight enzymes, of them, only *CS* and *IDH3A* were significant. *ALDH1A1* and *POU5F1/OCT4* negatively correlated with nine enzymes with significant negative correlation with CS2 and ME2 as well as *IDH3A,* respectively. Similarly, *CD133/PROM1* negatively correlated with nine enzymes with significance only with *ACO1* and *SUCLG2*. Consistently, *CD44* negatively correlated with eight enzymes with significance observed with *ACO2* and *MDH2*; whereas *NANOG* negatively correlated with five enzymes with significant negative correlation with *SUCLG2*. *NOTCH1* did not significantly correlate with any enzyme in the TCA cycle with a trend towards negative correlations with six enzymes (Figure S3).

These data indicate that the reliance of OCSCs cells on the TCA is demonstrated in TCGA data by the positive correlations between OCSC markers and TCA enzymes. Spheroids from characterized OVCAR3 not only exhibited significant upregulation of most of the TCA enzymes but also exhibited positive correlations with OCSC markers and TCA enzymes suggestive of their reliance on TCA cycle. The discrepancies between the two OCSC model systems further highlight the influence of environmental cues on the expression of their metabolic programing and plasticity.

Conversely, OCSC-like spheroids relied on anaerobic glycolysis and the PPP with decreased reliance on the TCA cycle compared to their parental cells [59]. Another study demonstrated that in SKOV3 and lung A549 model systems, cell populations with high telomerase activity, exhibited significantly higher ability to form spheroids (and hence CSC phenotype), and exhibited enhanced glycolysis, OXPHOS, and increased mitochondrial mass [60]. Consistently, a subset of cancer cells with stem like properties, called side population (SP) cells, were identified in OvCa and other cancer types and exhibited higher glycolytic activity than non-SP cells. The percentage of SP cells significantly increased in glucose-rich conditions. Paradoxically, glucose deprivation or the presence of a glycolytic inhibitor, 3-BrOP, significantly decreased the number of SP cells and decreased their tumor forming ability in mice xenografts [61]. Glucose-induced CSC-like SP proliferation was mediated through an ATP-dependent suppression of AMPK and activation of the AKT pathway [61].

### 3.4. OXPHOS in Ovarian Cancer Stem Cells

OXPHOS is a process by which NADH and FADH_2_ generated in TCA cycle transfer electrons to the mitochondrial ETC and occurs via several redox reactions that take place in the inner mitochondrial membrane (IMM). These reactions facilitate the generation of an electrochemical proton (H^+^) gradient, which subsequently drives the synthesis of energy rich adenosine triphosphate (ATP) by ATP synthases [62].

CSCs may exhibit highly glycolytic or OXPHOS phenotypes with plasticity of metabolic switch between phenotypes depending not only on cancer type and environmental cues, but also upon glucose starvation or OXPHOS blockade [55,63]. For example, CD44^+^/CD117^+^ OCSCs isolated from patients’ ascitic fluid exhibited enhanced glucose uptake with heightened OXPHOS. Upon glucose starvation, these OCSCs underwent complete quiescence and down-regulated most metabolic activities, while maintaining an OXPHOS profile [63]. In further support of this, cellular bio-energetic profiling of established and patient-derived OvCa cell lines showed that chemo-sensitive cancer cell lines displayed a glycolytic phenotype while their chemo-resistant counterparts developed an adaptive switch between OXPHOS and glycolysis [64]. Integrated proteomic, metabolomic, and bioenergetic analyses of OVTOKO cells growing in 3D spheroids and expressing high ALDH1A1 levels, showed a high-OXPHOS subtype relying on OXPHOS, supported by glutamine and FAO and a low-OXPHOS subtype that was mainly glycolytic [65].

**Figure 5:**
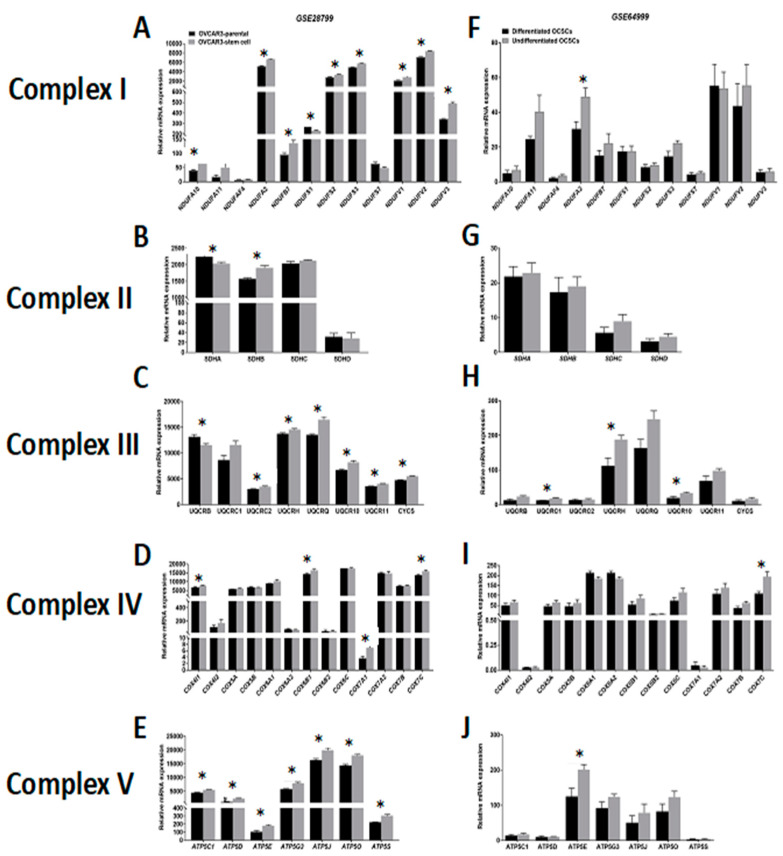
Expression of electron transport chain (ETC) complexes in *GSE28799* and *GSE64999.* Bars represent mean ± SEM of the relative mRNA expression of the indicated enzymes involved in complex I, (**A**–**F**); complex II, (**B**–**G**); complex III, (**C**–**H**); complex IV, (**D**–**I**), and complex V, (**E**–**J**) in OVCAR3 stem-cells vs. their parental cells in *GSE28799* and differentiated vs. undifferentiated OCSCs in *GSE64999*. Significance was determined using the multiple *t-*test and Holm–Sidak method with each row analyzed individually with *p* < 0.05, without assuming a consistent SD. * indicate the number of significant correlations either positive or negative.

**Figure 6:**
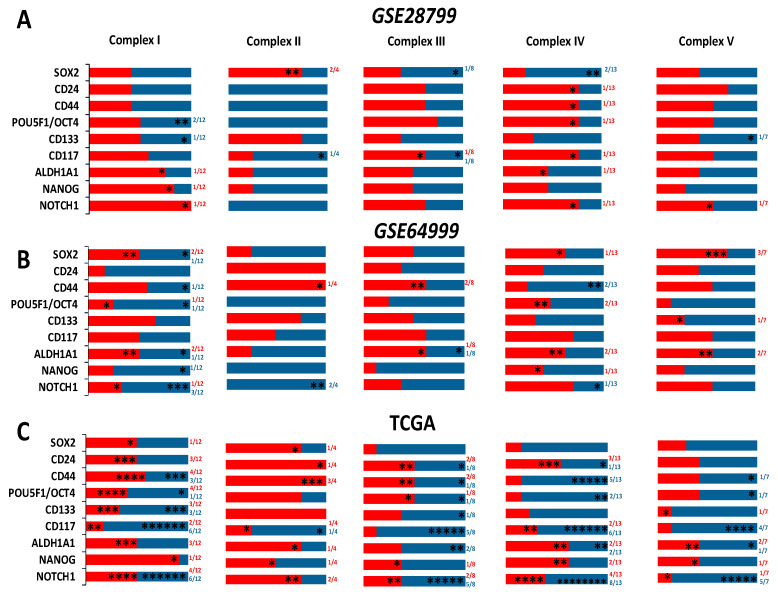
Correlations of the transcripts of OCSC markers and ETC complexes. Pearson Correlation between the expression of OCSC transcripts and the transcripts of the key enzymes involved in ETC in OVCAR3-OCSCs curated from *GSE28799* (**A**) and *GSE64999* (**B**), as well as patients’ tumors from TCGA (**C**). Bar graphs represent the prevalence of positive (**red**) and negative (**blue**) correlations of the indicated OCSC markers and ETC enzymes. Significance was determined using the multiple *t-*test and Holm–Sidak method with each row analyzed individually with *p* < 0.05, without assuming a consistent SD. * indicate the number of significant correlations either positive or negative.

Comparative analysis of the expression of ETC complexes in OVCAR3-OCSCs in *GSE28799* revealed significant upregulation of most of the transcripts of complex I (NADH:ubiquinone oxidoreductases, *NDUFA* Figure 5A), with inconsistent changes of enzymes of complex II (succinate dehydrogenases, *SDHA-D,* Figure 5B). Significant increase was observed in six out of eight transcripts of complex III enzymes (ubiquinol: cytochrome C oxidoreductases, *UQCR*) in OVCAR3-OCSCs compared to parental cells (Figure 5C), but only in four out of thirteen transcripts of complex IV enzymes (cytochrome C oxidases, *COX4I1, COX6B1, COX7A1,* and *COX7C,* Figure 5D). Whereas, OVCAR3-OCSCs exhibited significant increase in all the transcripts of complex V enzymes (ATP synthases) compared to parental cells (Figure 5E). In *GSE64999,* undifferentiated OCSCs exhibited significant upregulation only in one complex I enzyme, *NDUFA2,* compared to differentiated OCSCs (Figure 5F) with no significant changes in complex II enzymes (Figure 5G). In addition, undifferentiated OCSCs exhibited an upregulation trend of complex III enzyme transcripts with significance observed in three out of the eight enzymes investigated (Figure 5H), whereas for complex IV, they exhibited significant upregulation of *COX7C* with an upregulation trend in the other transcripts (Figure 5I). Concordantly, the transcripts of ATP synthases were upregulated in undifferentiated OCSCs with significance observed only in *ATP5E* (Figure 5J). Together, this data indicates that OCSC spheroids exhibit enrichment of the ETC mainly in complexes I, III, IV, and V, further supporting reliance on the OXPHOS phenotype. Interestingly, correlations between the expression of OCSC transcripts and the transcripts of the key ETC enzymes revealed that in OVCAR3-OCSCs in *GSE28799,* there was a trend of positive correlations with complex I enzymes with *NOTCH1, NANOG,* and *ALDH1A1* exhibiting significant correlations with the transcripts of one of the twelve enzymes. However, there were inconsistent positive and negative correlations between the transcripts of OCSC markers and the transcripts of complexes II, III, IV, and V (Figure 6A and Table S5). In contrast, in *GSE64999* the transcripts of OCSC markers in undifferentiated OCSCs exhibited inconsistent positive and negative correlations with transcripts of complexes I, III, IV, and V, with *CD24* and *CD44* exhibiting positive though insignificant correlations with complex II enzymes (Figure 6B and Table S6). Interestingly, the correlation patterns of the transcripts of OCSC markers and ETC enzymes in patients’ tumors in TCGA data (Figure 6C and Table 3) phenocopied those of undifferentiated OCSCs. These data suggest that OCSC markers do not correlate with those of ETC enzymes in the model systems or in patients’ tumors. The inconsistencies in the positive and negative correlations and their significance may be due to the existence of multiple isoforms for ETC enzymes with redundant activity, or compensation of the activity by mitochondrially encoded ETC enzymes.

Of note, is that analysis of OvCa cell line data curated from CCLE also revealed similar inconsistent pattern of positive and negative correlations with ETC enzymes (Figure S4). *CD44* exhibited negative correlations with all investigated enzymes of complexes I and V with significant negative correlations with *NDUFS1* and *NDUFS2* of complex I*. CD44* also exhibited negative correlations with four out of nine enzymes in complex III with significant negative correlation with *UQCRC2*. In addition, *CD44* exhibited negative though insignificant correlation with two out of four complex II enzymes and four out of thirteen complex IV enzymes. *SOX2* exhibited positive correlations with all ETC complexes with significant positive correlations with *NDUFS3* and *NDUFV1* of complex I, *CYSC* of complex III, and *ATP5J* of complex V. *POU5F1/OCT4* negatively correlated with most of the enzymes of complexes I-V with significant negative correlations with *NDUFA10*, *NDUFAF4,* and *NDUFAS1-3* of complex I, *SDHC* of complex II, *UQCRC1* and *UQCRH* of complex III, *COX5A* of complex IV as well as *ATP5D* of complex V. However, *POU5F1/OCT4* only positively correlated with *COX7A1* of complex IV. *PROM1/CD133* exhibited a trend of a negative correlations with complexes II, III, and V with significant negative correlation with *NDUFA10* of complex I and *UQCR10* of complex III as well as significant positive correlation with *COX4I1* and *COX6B1* of complex IV. *KIT/CD117* exhibited positive correlation with complex I, III, and V, and a negative correlation with complex IV, with a significant positive correlation only with *NDUFS1* of complex I. *ALDH1A1* exhibited negative correlation with complex I and II enzymes with significant negative correlation with *NDUFA10* of complex I, and *COX6A2* and *COX7A1* of complex IV. However, *ALDH1A1* positively correlated with *COX6A1.* Furthermore, *NANOG* exhibited positive correlation with complexes I, II, and V, with significant positive correlation only with *NDUFA11, SDHC,* and *ATP5J*. Finally, *NOTCH1* exhibited negative correlations with complexes II, IV, and V, with significant negative correlation only with *COX4I2* and *COX6B1* as well as *ATP5C1*. Together, data from CCLE further imply that the expression of the transcripts of OCSC markers *CD44, POU5F1/OCT4,* and *NOTCH1* exhibit most of the significant negative correlations.

### 3.5. Correlation of OCSC Markers With Lipid Metabolism

Lipid-associated pathways are essential for the maintenance of CSCs [66]. Lipid metabolism is inherently bound to the glucose and amino acid metabolic pathways in order to meet the increasing bio-energetic needs of CSCs. Specifically, epithelial OCSCs utilize OXPHOS and FAO to overcome glucose starvation, and this metabolic trait confers resistance to chemotherapy [64]. It has been shown that when CD44^+^CD117^+^ CSCs were isolated from OvCa primary culture, and detached from a monolayer into a suspension state, they reprogramed their metabolism from glycolysis to TCA cycle with active lipid metabolism compared to adherent cultures [67]. Consistently, ALDH^+^CD133^+^ OCSCs and spheroids have increased levels of unsaturated lipids compared to non-CSCs in monolayers [68]; an effect that was inhibited by inhibition of lipid desaturases that significantly reduced stemness and eliminated sphere formation *in vitro* and tumor initiation *in vivo* [68]. Moreover, overexpression of *fatty acid synthase* (*FASN*), a key enzyme in lipogenesis correlated with poor disease outcome [69]. Accordingly, the inhibition of *FASN* reversed platinum resistance in resistant OvCa cells [70].

**Figure 7:**
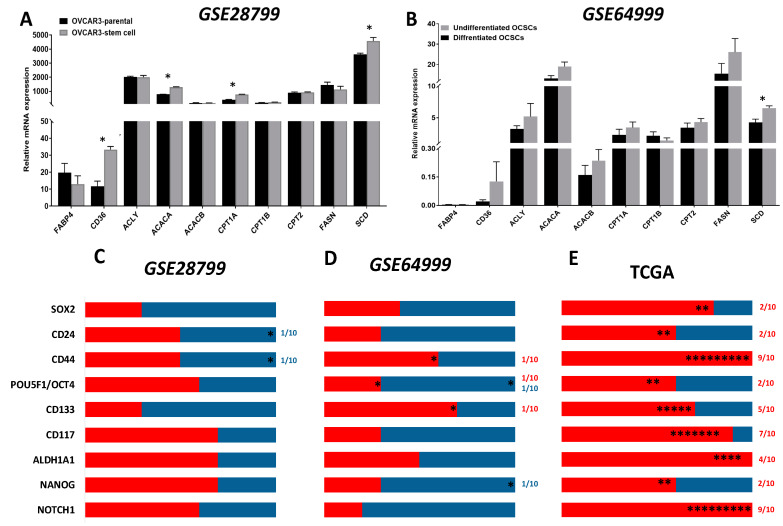
Expression of transporters and enzymes of lipid metabolism in OCSC models. (**A**,**B**) Bars represent mean ± SEM of the relative mRNA expression of the indicated transporters and lipid metabolism enzymes in OVCAR3 stem-cells vs. their parental cells in *GSE28799* and undifferentiated vs. differentiated OCSCs in *GSE64999.* Significance was determined using the multiple *t*-test and Holm–Sidak method with each row analyzed individually with *p* < 0.05, without assuming a consistent SD. (**C**–**E**) Bar graphs represent the prevalence of positive (**red**) and negative (**blue**) correlations of the indicated OCSC markers and lipid metabolism enzymes in Supplement Tables S7–S8 and Table 4. * indicate the number of significant correlations either positive or negative.

Analysis of OVCAR3-OCSCs transcripts in *GSE28799* dataset, revealed that compared to parental OVCAR3, OVCAR3-OCSCs exhibited significant increase in the transcripts of fatty acid transporter (*CD36), acetyl CoA carboxylase A (ACACA)* that catalyzes the first committed step in fatty acid (FA) synthesis from acetyl CoA to malonyl CoA [71], *stearoyl-CoA desaturase* (*SCD*), that is also involved in FA biosynthesis, mainly unsaturated FAs, as well as *carnitine palmitoyltransferase 1A (CPT1A)* that catalyzes the first committed step in FAO [72,73] (Figure 7A). Analysis of the transcripts in undifferentiated vs. differentiated OCSC models in *GSE64999* dataset revealed a significant increase in *SCD* in the undifferentiated OCSCs with an increase, though insignificant, in the enzymes involved in FA synthesis and oxidation as well as *CD36* (Figure 7B). This data indicates that OCSCs exhibit a lipid metabolic phenotype that is consistent with the OvCa in the lipid-rich peritoneal TME.

Correlation of the putative OCSC markers and lipid transporters and enzymes in OVCAR3-OCSCs in *GSE28799* dataset revealed that *NOTCH1, NANOG, OCT4, ALDH1A1,* and *CD117* exhibited more positive, though insignificant, correlations with lipid metabolism markers than *CD44, CD24, CD133,* or *SOX2.* The latter markers exhibited a trend towards negative correlations, with *CD44* and *CD24* exhibiting significant negative correlations with fatty acid-binding protein 4 *(FABP4)* (Figure 7C and Table S7). In *GSE64999*, *CD44, CD133*, and *ALDH1A1* exhibited more positive correlations with lipid metabolic enzymes with significance between *CD44* and *CPT1B* as well as *CD133* and *CPT1A*. *OCT4* exhibited significant positive correlation with *CD36,* but negatively correlated with *ACACA*. *NANOG* was negatively correlated with *ACACA* (Figure 7D and Table S8). TCGA data indicated significant positive correlation between *NOTCH1* and *CD44,* with lipid metabolic markers, followed by *CD133, ALDH1A1,* and *CD117* with significant positive correlations exhibited by *SOX2* (Figure 7E and Table 4). Conversely, in CCLE datasets, *CD44* negatively correlated with *ACACB, CPT1A,* and *CPT2*. *CD117* positively correlated with *ACACB, CPT2, FASN,* and *SCD1*. *CD133* positively correlated with *CPT1A*. *ALDH1A1* was negatively correlated with *ACACB*. *SOX2* was positively correlated with *CPT1A* and *CPT2* and negatively correlated with *FABP4*. *OCT4* was negatively correlated with *FABP4, ATP citrate lyase (ACLY), ACACA, FASN,* and *SCD1* and positively correlated with *CPT1A/B*. *NANOG* was positively correlated with *CPT2*. *NOTCH1* was not significantly correlated with the enzymes investigated (Figure S5). These data further support the distinct metabolic phenotypes and “metabolic plasticity” of OCSCs as the acquisition of the OCSC phenotype is associated with enrichment of a lipid metabolic signature. These may explain the ability of OCSCs to survive in the lipid-rich peritoneal TME leading to recurrence after optimal or suboptimal surgery and chemotherapy.

### 3.6. Glutamine/Glutamate Metabolism in OCSCs

Glutamine metabolism provides the carbon and amino-nitrogen that are necessary for biosynthesis of amino acids, nucleotides, and lipids [74,75,76]. As an anabolic process, glutaminolysis promotes the production of macromolecules with lower energetic potential [77]. Furthermore, glutamine enters cancer cells through the *alanine, serine, cysteine transporter 2* (*ASCT2*; also known as *SLC1A5*) and is subsequently hydrolyzed to glutamate and ammonia by *glutaminase* (*GLS*). Glutamate has dual roles: while it can be combined with cysteine and glycine to form the reduced glutathione (GSH), a major antioxidant that regulates oxidative stress [78], it can also be converted into α-ketoglutarate (αKG) by *glutamate dehydrogenases* (*GLUD*) to provide TCA cycle intermediates and, hence, energy production [79]. Furthermore, OCSCs like other CSCs exploit glutamine metabolism for therapeutic resistance. Glutaminolysis significantly correlated with poor survival of OvCa patients [80]. Additionally, glutamine and glutamate concentrations were higher in spheroids than in adherent OCSCs [65]. Targeting glutamine metabolism using a pan-transaminase inhibitor hindered the growth of spheroids from ovarian clear cell carcinomas with inhibition of the mTOR pathway [65], suggesting a promising therapeutic strategy.

**Figure 8:**
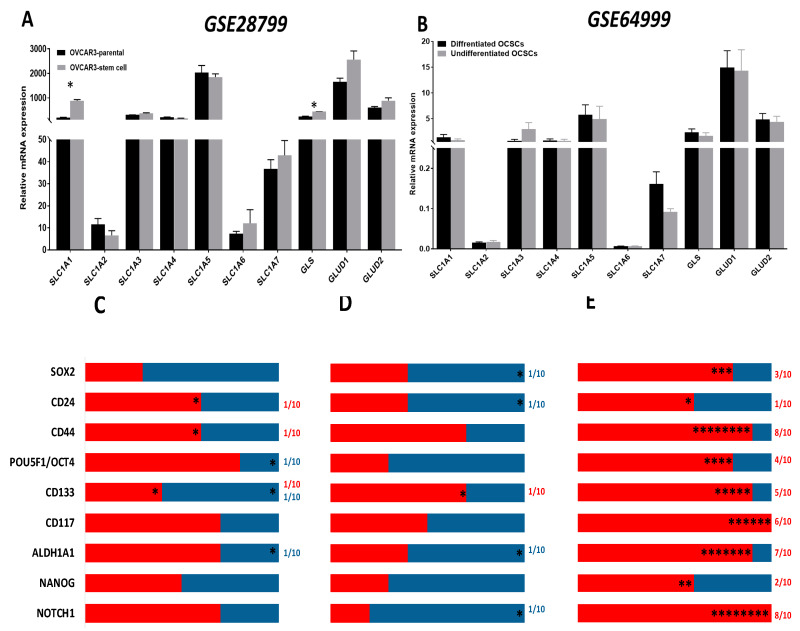
Expression of transporters and enzymes of glutamine/glutamate metabolism in OCSC models. (**A**,**B**) Bars represent mean ± SEM of the relative mRNA expression of the indicated transporters and glutamine/glutamate metabolism enzymes in OVCAR3 stem-cells vs. their parental cells in *GSE28799* and undifferentiated vs. differentiated OCSCs in *GSE64999.* Significance was determined using the multiple *t*-test and Holm–Sidak method with each row analyzed individually with *p* < 0.05, without assuming a consistent SD. (**C**–**E**) Bar graphs represent the prevalence of positive (**red**) and negative (**blue**) correlations of the indicated OCSC markers and glutamine/glutamate metabolism enzymes in Supplement Tables S9–S10 and Table 5. * indicate the number of significant correlations either positive or negative.

Analysis of the expression of the enzymes involved in glutamine metabolism in OCSCs and their parental cells in *GSE28799* revealed that the glutamine transporter *SLC1A1* and the enzyme *GLS,* were significantly upregulated in OVCAR3 spheroid-derived OCSCs compared to their parental OVCAR3 cells (Figure 8A). In contrast, there was no significant difference in glutamine metabolism enzymes between undifferentiated and differentiated spheroids in *GSE64999* (Figure 8B). In both datasets*,* very few correlations between stem cell markers and glutamine transporters were noted. In *GSE28799*, both *CD24* and *CD44* positively correlated with *SLC1A1*, while *CD133* positively correlated with *GLS* and negatively correlated with *GLUD2*. *ALDH1A1* positively correlated with *SLC1A2* while *OCT4* positively correlated with *SLC1A3* (Figure 8C and Table S9). In *GSE64999, CD133* positively correlated with *GLS*. *CD24* negatively correlated with *GLUD1*. *ALDH1A1* and *SOX2* both negatively correlated with *SLC1A6*. *NOTCH1* negatively correlated with *SLC1A1* (Figure 8D and Table S10).

Further analysis of patients’ tumors from TCGA data indicated that *CD44, ALDH1A1,* and *NOTCH1* exhibited significantly positive correlations with enzymes involved in glutamine metabolism. *CD44* positively correlated with *SLC1A1-5*, *GLS*, and *GLUD1/2*. Similarly, *ALDH1A1* exhibited significant positive correlation with four out of the seven glutamine transporters, along with *GLS* and *GLUD1/2*. *NOTCH1* significantly positively correlated with five out of the seven glutamine transporters, along with *GLS* and *GLUD1/2. CD133* positively correlated with five out of the seven glutamine transporters, while *CD117* correlated with three out of seven glutamine transporters, as well as with *GLS* and *GLUD1/2*. *SOX2* and *OCT4* positively correlated with three and two glutamine transporters, respectively. *OCT4* also positively correlated with *GLS* and *GLUD1/2*. *CD24* positively correlated with one glutamine transporter, as well as *SLC1A1*, but no other significant correlations with other glutamine transporters. *NANOG* was not positively correlated with any of the glutamine transporters yet was correlated with *GLS* and *GLUD2* (Figure 8E and Table 5). Of note is that transcriptomic profiling of data of the OvCa cell lines in CCLE demonstrated that the transcripts of *CD44* and *OCT4* both positively correlated with *SLC1A1* and *GLS;* whereas *OCT4* negatively correlated with *SLC1A2* and *SLC1A5*. *CD117* positively correlated with *SLC1A3,* while *CD133* positively correlated with *SLC1A5*. Additionally, *ALDH1A1* negatively correlated with *SLC1A7,* while *NANOG* negatively correlated with *SLC1A1* (Figure S6). In summary, data from TCGA show positive correlations between the transcripts of OCSC markers and glutamine metabolism enzymes, whereas in OCSC experimental models of *GSE64999, GSE28799,* and *CCLE,* the number of positive and negative correlations observed was significantly lower implying that glutamine metabolism is indispensable for OCSCs and OvCa cells grown in normal conditions, whereas in their complex TME, OvCa cells utilize glutamine metabolism in an anaplerotic reaction for generating energy and metabolic intermediates to fulfill their increasing demands.

While our findings are based on OCSC transcript levels, it is necessary to clarify that the transcript level and proteome levels may not necessarily correlate due to post transcriptional modifications, including splice variation. Alternative splicing accounts for the major difference between the number of protein-coding genes and the number of proteins that are ultimately produced from translation [81]. Through the various splicing mechanisms, as constitutive splicing, mutually exclusive exons, cassette alternative exon, alternative 3′ or 5′ splice site, and intron retention, splicing plays a key role in protein diversification. In effect, by allowing several functionally distinctive proteins to be encoded by the same original gene, splicing does not allow for the direct conversion of the transcriptomic signature into the proteomic signature [81]. Moreover, due to intra-clonal heterogeneity, it is challenging to assign a specific set of stemness markers that define the different subsets of CSCs [82]. Such heterogeneity includes quiescent, precancerous, primary, migrating, chemo-resistant, and radio-resistant CSCs [83]. In OCSCs specifically, multiple subtypes have been identified including proliferating CSCs expressing *OCT4*, *ALDH1/2*, *CD44*, and *LGR5/*Ki67^+^ markers and non-proliferating CSCs expressing *SSEA4*^+^/Ki67^−^ or *ALDH1/2*^+^/Ki67^−^ markers. Other subsets of OCSCs include *CD133*^+^ and *CD44*^+^/*CD117*^+^ all of which can be influenced by tumor microenvironmental cues, upregulation of signaling pathways, and tumor grade, making it difficult to confine OCSCs to a standardized list of markers [4,82].

## 4. Conclusions

In this study, we sought to investigate the predictive significance of the gene expression of OCSCs in correlation with transcripts of metabolic pathways that are upregulated in HGSOC, that could inform about the metabolic plasticity of tumors, predict the behavior of recurrent or chemo-resistant disease, and ultimately may guide on therapeutics targeting the enriched pathways in combination with standard of care therapy. Comparison of transcripts of OCSC markers with those of enzymes involved in metabolic pathways in OCSC spheroid model systems with patients’ data revealed several distinctive differences in the expression of the stem cell markers. Correlations with energy metabolic pathways, not only between the two model systems investigated, but also with patients’ tumors and cumulative behavior of OvCa cell data from CCLE were elucidated. While these differences between spheroid model systems could be attributed to technical variability in establishing and maintaining of OCSCs as spheroids, nevertheless, they highlight the significance of OvCa heterogeneity, and the influence of micro-environmental cues on metabolic plasticity of OCSCs that may influence their survival and self-renewal capabilities.

It is noteworthy that the limitation of the present study is the paucity of data from established model systems of OCSCs with adequate biological replica that can be used for systematic biological, metabolic as well as transcriptomic analyses to bridge the gap of metabolic programing in OCSCs.

## Figures and Tables

**Table 1:** Pearson Correlation between the expression of CSC transcripts and the transcripts of glucose transporters and the key enzymes involved in glycolysis in ovarian cancer specimens from TCGA and curated from GEPIA. Red indicates significant positive and blue indicates negative correlations.

TCGA	CD44		CD24		CD117		CD133		ALDH1A1		SOX2		4-Oct		NANOG		NOTCH1	
	Pearson R	*p*-Value	Pearson R	*p*-Value	Pearson R	*p*-Value	Pearson R	*p*-Value	Pearson R	*p*-Value	Pearson R	*p*-Value	Pearson R	*p*-Value	Pearson R	*p*-Value	Pearson R	*p*-Value
**HK1**	0.29	0.00	7e−04	0.99	0.15	0.00	0.14	0.00	0.13	0.01	0.03	0.60	0.03	0.61	0.06	0.25	0.40	0.00
**HK2**	0.13	0.01	0.09	0.08	0.20	2.9e−05	0.14	0.00	0.09	0.07	0.01	0.82	0.11	0.03	0.03	0.60	0.30	4.9e−10
**SLC2A1**	0.09	0.07	0.00	0.95	0.23	1.7e−06	0.04	0.42	0.10	0.03	0.00	0.87	0.00	0.95	0.03	0.60	0.22	2.9e−06
**SLC2A2**	0.07	0.16	−0.03	0.60	0.12	0.02	−0.04	0.42	0.08	0.09	−0.03	0.48	−0.04	0.43	0.01	0.77	0.19	0.00
**SLC2A3**	0.31	9.7e−11	−0.02	0.68	0.19	0.00	0.00	0.95	0.07	0.15	0.11	0.02	−0.03	0.54	0.03	0.49	0.24	3.3e−07
**SLC2A4**	0.01	0.77	0.04	0.37	0.19	9.4e−05	0.01	0.87	0.00	0.97	0.09	0.06	0.01	0.77	0.09	0.06	0.18	0.00
**SLC2A5**	0.45	0.00	0.02	0.74	0.06	0.24	0.00	0.98	−0.03	0.61	−0.03	0.57	0.03	0.52	−0.05	0.29	0.18	0.00
**SLC2A6**	0.24	5e−07	−0.12	0.01	0.00	0.90	0.00	0.89	0.05	0.31	0.00	0.86	−0.04	0.40	−0.05	0.28	0.29	1e−09
**PGK1**	0.30	4.4 × 10^10^	−0.01	0.82	0.02	0.63	0.02	0.69	0.19	8.7e−05	0.10	0.04	0.00	1.00	0.04	0.45	0.14	0.00
**PFKFB3**	0.35	0.00	0.00	0.93	0.11	0.02	0.11	0.03	0.04	0.39	0.10	0.03	0.09	0.07	−0.01	0.77	0.28	6.9e−09
**LDHA**	0.20	3.5e−05	−0.03	0.60	−0.02	0.74	−0.03	0.60	0.13	0.01	0.06	0.24	0.04	0.46	0.00	0.97	−0.02	0.65
**FBP1**	0.40	0.00	−0.11	0.02	−0.06	0.24	0.00	0.98	0.11	0.03	0.06	0.19	0.00	0.96	0.00	0.93	0.03	0.48
**PDK1**	0.12	0.01	0.02	0.66	0.04	0.38	0.06	0.23	0.24	3.9e−07	0.04	0.41	0.04	0.42	0.01	0.80	0.13	0.01
**PDHA**	0.12	0.01	−0.02	0.75	−0.02	0.70	0.07	0.13	0.14	0.01	0.09	0.06	0.08	0.11	0.03	0.51	0.22	5.4e−06
**TPI1**	0.07	0.15	0.05	0.33	−0.04	0.47	−0.06	0.25	0.03	0.56	0.02	0.75	−0.01	0.77	0.08	0.10	−0.02	0.74
**ENO1**	0.23	1.3e−06	0.07	0.17	−0.02	0.71	0.09	0.08	0.10	0.05	0.01	0.86	0.13	0.01	−0.04	0.47	0.12	0.02
**PHGDH**	0.02	0.71	0.03	0.50	0.19	5.3e−05	0.10	0.05	0.20	4.8e−05	0.22	6.4e−06	−0.05	0.30	0.14	0.00	0.14	0.00

**Table 2:** Correlation of OCSC markers with TCA enzymes in TCGA. Pearson Correlation between the expression of CSC transcripts and the transcripts of the key TCA enzymes in ovarian cancer specimens from TCGA and curated from GEPIA. Red indicates significant positive, blue indicates negative correlations, and black indicates insignificant *p*-values of either positive or negative correlations.

TCGA	CD44		CD24		CD117		CD133		ALDH1A1		SOX2		4-Oct		NANOG		NOTCH1	
	Pearson R	*p*-Value	Pearson R	*p*-Value	Pearson R	*p*-Value	Pearson R	*p*-Value	Pearson R	*p*-Value	Pearson R	*p*-Value	Pearson R	*p*-Value	Pearson R	*p*-Value	Pearson R	*p*-Value
**CS**	0.19	8.2e−05	0.16	0.00	0.23	9.9e−07	0.09	0.06	0.15	0.00	0.00	0.96	0.07	0.18	0.11	0.02	0.39	0.00
**ACO1**	0.30	1.6e−10	0.08	0.09	0.21	1.1e−05	0.24	7.6e−07	0.30	5.1e−10	0.10	0.05	0.11	0.02	0.00	0.99	0.34	2.8e−13
**ACO2**	0.18	0.00	0.11	0.02	−0.06	0.17	0.04	0.41	−0.03	0.58	−0.03	0.58	0.10	0.05	−0.04	0.45	0.24	3.6e−07
**IDH1**	0.12	0.01	0.08	0.11	0.01	0.77	0.06	0.21	0.16	0.00	0.09	0.08	0.01	0.88	0.02	0.63	0.17	0.00
**IDH2**	0.16	0.00	0.07	0.13	0.11	0.03	0.07	0.14	0.09	0.08	0.05	0.27	−0.03	0.51	0.04	0.39	0.25	2.6e−07
**IDH3A**	0.16	0.00	−0.09	0.06	−0.04	0.44	0.07	0.15	0.09	0.06	0.04	0.37	−0.03	0.58	−0.01	0.77	0.24	4.2e−07
**IDH3B**	−0.08	0.12	0.09	0.06	0.09	0.07	0.04	0.48	0.03	0.60	−0.05	0.35	0.06	0.21	0.04	0.47	0.24	6e−07
**SUCLG1**	0.13	0.01	0.16	0.00	0.01	0.86	0.01	0.81	0.13	0.01	0.00	0.90	0.00	0.99	0.03	0.50	0.04	0.41
**SUCLG2**	0.19	9.3e−05	0.01	0.89	−0.08	0.12	0.05	0.27	0.06	0.22	0.08	0.12	0.11	0.02	0.00	0.90	0.05	0.28
**MDH2**	0.20	4.4e−05	0.00	0.92	−0.1	0.05	0.00	0.96	0.00	0.97	0.01	0.79	0.07	0.17	0.01	0.87	0.10	0.04
**ME2**	0.27	9.6e−09	0.16	0.00	0.05	0.33	0.08	0.09	0.09	0.07	0.04	0.45	0.13	0.01	−0.03	0.65	0.12	0.01

**Table 3:** Pearson correlation between the expression of CSC transcripts and the transcripts of the key enzymes involved in the ETC in patients’ data curated from TCGA. Red indicates significant positive, blue indicates negative correlations, and black indicates insignificant *p*-values of either positive or negative correlations.

TCGA																		
	CD44		CD24		CD117		CD133		ALDH1A1		SOX2		4-Oct		NANOG		NOTCH1	
Complex I	Pearson R	*p*-Value	Pearson R	*p*-Value	Pearson R	*p*-Value	Pearson R	*p*-Value	Pearson R	*p*-Value	Pearson R	*p*-Value	Pearson R	*p*-Value	Pearson R	*p*-Value	Pearson R	*p*-Value
NDUFA10	0.04	0.44	0.02	0.72	−0.02	0.72	−0.03	0.52	0.09	0.06	−0.02	0.62	−0.03	0.53	0.00	0.93	0.10	0.05
NDUFA11	−0.02	0.62	0.08	0.10	0.00	0.93	−0.05	0.29	−0.01	0.86	−0.02	0.67	−0.07	0.16	0.05	0.30	−0.15	0.00
NDUFAF4	−0.12	0.01	0.23	1.6e−06	−0.06	0.22	−0.09	0.05	0.03	0.54	−0.02	0.76	0.00	1.00	0.05	0.29	−0.13	0.01
NDUFA2	−0.13	0.01	−0.03	0.48	−0.19	1e−04	−0.11	0.02	−0.02	0.66	−0.02	0.64	−0.09	0.06	0.01	0.83	−0.29	1.7e−09
NDUFB7	−0.12	0.01	−0.02	0.67	−0.15	0.00	−0.09	0.06	−0.05	0.32	−0.04	0.40	−0.07	0.15	0.03	0.49	−0.18	0.00
NDUFS1	0.20	2.8e−05	0.16	0.00	0.14	0.00	0.14	0.00	0.17	0.00	0.02	0.69	0.07	0.14	0.03	0.57	0.39	0.00
NDUFS2	0.18	0.00	0.13	0.01	0.15	0.00	0.13	0.01	0.06	0.23	0.09	0.06	0.15	0.00	0.16	0.00	0.36	1.3e−14
NDUFS3	0.12	0.01	−0.05	0.29	−0.12	0.01	−0.02	0.74	0.00	0.93	0.04	0.39	0.04	0.39	0.02	0.62	−0.10	0.05
NDUFS7	0.15	0.00	−0.02	0.73	−0.18	0.00	−0.08	0.10	−0.03	0.49	−0.01	0.89	−0.01	0.86	−0.02	0.69	−0.11	0.02
NDUFV1	0.05	0.33	−0.02	0.71	−0.13	0.01	−0.01	0.80	−0.04	0.43	0.03	0.51	0.18	0.00	0.03	0.61	0.08	0.11
NDUFV2	−0.02	0.71	0.05	0.29	−0.11	0.02	0.15	0.00	0.02	0.65	0.06	0.18	−1e−04	1.00	0.05	0.35	0.01	0.88
NDUFV3	0.06	0.24	−0.01	0.84	−0.03	0.56	0.07	0.14	0.09	0.07	0.03	0.52	0.10	0.03	0.08	0.10	0.15	0.00
**Complex II**																		
SDHA	0.11	0.03	0.03	0.52	−0.04	0.46	0.05	0.35	0.03	0.52	0.11	0.03	0.06	0.20	0.04	0.39	0.16	0.00
SDHB	0.18	0.00	0.09	0.07	−0.05	0.28	0.05	0.29	−0.02	0.76	−0.05	0.29	0.08	0.10	−0.03	0.56	0.03	0.54
SDHC	0.16	0.00	0.11	0.03	0.10	0.04	0.07	0.15	0.21	1.4e−05	0.06	0.23	−0.0029	0.95	0.11	0.03	0.11	0.02
SDHD	0.03	0.59	0.08	0.09	−0.12	0.01	0.02	0.72	0.00	0.96	0.02	0.75	0.04	0.46	−0.04	0.43	−0.03	0.48
**Complex III**																		
UQCRB	−0.12	0.02	−0.06	0.19	−0.20	2.6e−05	−0.08	0.09	−0.14	0.00	−0.06	0.20	−0.1	0.04	−0.04	0.47	−0.20	2.1e−05
UQCRC1	0.03	0.54	0.08	0.09	0.00	0.94	0.06	0.25	0.08	0.09	0.04	0.39	0.06	0.20	0.13	0.01	0.17	0.00
UQCRC2	0.21	1.3e−05	0.17	0.00	0.05	0.31	0.07	0.17	0.00	0.92	−0.06	0.23	0.15	0.00	0.04	0.37	0.07	0.16
UQCRH	−0.04	0.42	−0.13	0.01	−0.13	0.01	−0.01	0.82	−0.01	0.84	0.00	1.00	−0.03	0.58	−0.08	0.12	−0.17	0.00
UQCRQ	−0.05	0.34	−0.06	0.20	−0.17	0.00	−0.06	0.19	−0.03	0.60	−0.02	0.70	−0.01	0.77	−0.04	0.41	−0.31	3.6e−11
UQCR10	0.04	0.43	0.15	0.00	−0.1	0.04	0.03	0.50	0.00	0.97	−0.02	0.70	0.06	0.23	−0.02	0.66	−0.11	0.02
UQCR11	−0.03	0.57	−0.05	0.32	−0.21	9.6e−06	−0.10	0.04	−0.07	0.18	−0.03	0.50	−0.1	0.04	−0.03	0.59	−0.29	8e−10
CYCS	0.09	0.05	0.07	0.18	−0.06	0.20	−0.04	0.43	0.09	0.06	−0.02	0.73	0.02	0.64	0.03	0.60	0.11	0.03
**Complex IV**																		
COX4I1	0.01	0.81	0.10	0.05	−0.15	0.00	0.03	0.57	−0.09	0.07	−0.06	0.26	0.03	0.50	−0.06	0.21	−0.19	1e−04
COX4I2	−0.07	0.18	0.06	0.22	0.25	2.2e−07	0.01	0.83	0.17	0.00	−0.01	0.82	−0.08	0.10	0.16	0.00	0.15	0.00
COX5A	0.00	0.95	−0.09	0.08	−0.19	9.3e−05	−0.04	0.42	0.00	0.95	−0.04	0.43	−0.04	0.42	−0.05	0.31	−0.13	0.01
COX5B	−0.22	6.6e−06	0.10	0.04	−0.18	0.00	−0.07	0.13	−0.09	0.08	−0.02	0.64	−0.02	0.68	−0.04	0.36	−0.27	1.7e−08
COX6A1	−0.1	0.04	0.03	0.53	−0.11	0.03	−0.06	0.23	0.07	0.13	0.01	0.87	−0.10	0.04	0.06	0.19	−0.12	0.02
COX6A2	−0.13	0.01	0.09	0.06	0.20	3.1e−05	0.00	0.92	0.14	0.00	0.02	0.69	−0.07	0.14	0.24	3.6e−07	0.04	0.39
COX6B1	−0.06	0.21	−0.03	0.61	−0.02	0.73	−0.06	0.23	0.07	0.15	−0.01	0.87	0.01	0.85	0.07	0.15	−0.19	0.00
COX6B2	−0.04	0.38	−0.01	0.77	0.06	0.20	−0.03	0.54	0.00	0.94	−0.03	0.48	0.00	0.96	0.07	0.15	0.23	2.6e−06
COX6C	−0.09	0.05	−0.12	0.01	−0.20	5e−05	−0.032	0.51	−0.13	0.01	−0.04	0.39	−0.12	0.01	−0.01	0.91	−0.12	0.01
COX7A1	−0.02	0.64	−0.06	0.24	0.13	0.01	−0.03	0.56	0.08	0.09	−0.04	0.38	−0.06	0.19	0.02	0.67	0.00	0.99
COX7A2	−0.08	0.09	0.09	0.05	−0.04	0.40	−0.09	0.06	0.02	0.74	0.00	0.93	−0.08	0.09	0.01	0.87	−0.19	5.8e−05
COX7B	−0.02	0.70	−0.01	0.78	−0.19	1e−04	−0.07	0.14	0.01	0.78	0.00	0.95	−0.07	0.15	0.00	0.98	−0.27	1.1e−08
COX7C	−0.21	9.9e−06	0.03	0.55	−0.08	0.09	−0.09	0.08	−0.10	0.04	−0.09	0.06	0.00	0.96	−0.01	0.84	−0.30	2.8e−10
**Complex V**																		
ATP5C1	0.03	0.51	0.08	0.11	−0.06	0.24	−0.06	0.22	−0.02	0.62	0.01	0.87	−0.05	0.36	−0.05	0.28	−0.07	0.13
ATP5D	−0.02	0.63	−0.01	0.91	−0.22	7.6e−06	−0.11	0.02	−0.15	0.00	−0.06	0.26	0.00	0.93	−0.04	0.38	−0.18	0.00
ATP5E	−0.14	0.00	0.00	0.93	−0.05	0.33	−0.03	0.54	0.03	0.52	0.06	0.26	−0.10	0.04	0.02	0.76	−0.19	0.00
ATP5G3	0.07	0.14	−0.04	0.36	−0.16	0.00	−0.08	0.08	−0.01	0.87	−0.06	0.23	−0.05	0.34	−0.07	0.17	−0.13	0.01
ATP5J	−0.03	0.52	−0.02	0.65	−0.10	0.05	−0.04	0.44	0.17	0.00	−0.01	0.84	0.01	0.89	0.00	0.97	−0.17	0.00
ATP5O	−0.10	0.04	−0.02	0.75	−0.10	0.03	−0.06	0.23	−0.07	0.14	−0.07	0.14	0.06	0.25	0.04	0.46	−0.19	0.00
ATP5S	0.02	0.71	0.00	0.99	0.08	0.10	0.10	0.03	0.14	0.00	−0.01	0.78	0.05	0.32	0.13	0.01	0.10	0.04

**Table 4:** Correlation between OCSC markers and lipid transporters and key enzymes in lipid metabolism in TCGA. Red indicates significant positive, and black indicates insignificant *p*-values of either positive or negative correlations.

TCGA	CD44		CD24		CD117		CD133		ALDH1A1		SOX2		4-Oct		NANOG		NOTCH1	
	Pearson R	*p*-Value	Pearson R	*p*-Value	Pearson R	*p*-Value	Pearson R	*p*-Value	Pearson R	*p*-Value	Pearson R	*p*-Value	Pearson R	*p*-Value	Pearson R	*p*-Value	Pearson R	*p*-Value
**FABP4**	0.04	0.43	−0.04	0.44	0.05	0.28	−0.07	0.13	0.05	0.33	−0.04	0.43	0.00	0.87	−0.06	0.21	0.07	0.13
**CD36**	0.17	0.00	−0.02	0.63	0.11	0.02	−0.07	0.14	0.09	0.07	0.03	0.51	−0.03	0.55	0.00	0.90	0.24	4e−07
**ACLY**	0.21	9e−06	0.13	0.01	0.37	3.1e−15	0.21	1.8e−05	0.15	0.00	0.04	0.43	0.01	0.85	0.12	0.02	0.51	0.00
**ACACA**	0.16	0.00	0.20	3.1e−05	0.19	0.00	0.22	4.5e−06	0.10	0.03	0.01	0.79	0.08	0.11	0.02	0.73	0.32	6.3e−12
**ACACB**	0.10	0.04	0.00	0.99	0.27	1.7e−08	0.11	0.02	0.13	0.01	0.03	0.58	0.10	0.04	0.12	0.02	0.40	0.00
**CPT1A**	0.12	0.01	0.03	0.56	0.11	0.02	0.16	0.00	0.09	0.07	0.06	0.20	0.09	0.07	0.00	0.89	0.35	7.1e−14
**CPT1B**	0.16	0.00	−0.07	0.15	−0.05	0.30	−0.02	0.64	0.00	0.93	0.12	0.02	0.13	0.01	0.08	0.11	0.17	3e−04
**CPT2**	0.19	7.7e−05	0.03	0.57	0.08	0.08	0.18	0.00	0.18	0.00	0.09	0.06	0.06	0.20	0.09	0.07	0.29	7.1e−10
**FASN**	0.08	0.08	0.06	0.25	0.10	0.03	0.05	0.31	0.05	0.28	0.00	0.85	−0.02	0.66	0.02	0.67	0.42	0.00
**SCD1**	0.27	1.3e−08	0.06	0.20	0.11	0.02	0.08	0.10	0.08	0.12	0.11	0.03	−0.05	0.28	−0.02	0.67	0.27	1.5e−08

**Table 5:** Correlation between OCSC markers and glutamate transporters and key enzymes in glutamate/glutamine metabolism in TCGA. Red indicates significant positive and black indicates insignificant *p*-values of either positive or negative correlations.

TCGA	CD44		CD24		CD117		CD133		ALDH1A1		SOX2		4-Oct		NANOG		NOTCH1	
	Pearson R	*p*-Value	Pearson R	*p*-Value	Pearson R	*p*-Value	Pearson R	*p*-Value	Pearson R	*p*-Value	Pearson R	*p*-Value	Pearson R	*p*-Value	Pearson R	*p*-Value	Pearson R	*p*-Value
**SLC1A1**	0.32	1.2e−11	0.10	0.05	0.04	0.39	0.20	3.9e−05	0.12	0.01	0.15	0.00	0.18	0.00	−0.04	0.40	0.09	0.06
**SLC1A2**	0.16	0.00	0.00	0.93	0.25	1e−07	0.12	0.01	0.22	4.1e−06	0.05	0.31	0.04	0.44	0.07	0.13	0.14	0.00
**SLC1A3**	0.20	4.6e−05	0.08	0.12	0.09	0.08	0.18	0.00	−0.04	0.47	0.08	0.11	0.20	4.8e−05	−0.01	0.82	0.15	0.00
**SLC1A4**	0.27	1.5e−08	−0.03	0.60	0.27	1.8e−08	0.13	0.01	0.25	2.3e−07	0.11	0.02	0.00	0.99	0.09	0.06	0.36	1.4e−14
**SLC1A5**	0.24	3.7e−07	−0.08	0.10	0.06	0.21	0.03	0.56	0.05	0.35	0.15	0.00	0.03	0.50	−0.03	0.58	0.22	7.4e−06
**SLC1A6**	−0.02	0.64	0.02	0.76	0.03	0.53	0.02	0.69	0.00	0.96	0.01	0.78	−0.04	0.38	0.06	0.21	0.04	0.40
**SLC1A7**	0.09	0.08	−0.06	0.21	0.43	0.00	0.25	2.4e−07	0.16	0.00	−0.05	0.35	−0.03	0.50	−0.04	0.47	0.33	2e−12
**GLS**	0.15	0.00	−0.01	0.86	0.11	0.02	−0.01	0.86	0.18	0.00	0.03	0.49	0.05	0.30	0.13	0.01	0.36	2.8e−14
**GLUD1**	0.10	0.03	0.02	0.73	0.11	0.03	0.06	0.20	0.11	0.02	−0.02	0.68	0.14	0.00	0.05	0.35	0.25	2.2e−07
**GLUD2**	0.19	7.8e−05	0.04	0.73	0.25	2.6e−07	0.05	0.31	0.26	4.8e−08	0.09	0.08	0.15	0.00	0.31	9.3e−11	0.27	1.3e−08

## Supplementary Materials

The following are available online at https://www.mdpi.com/2072-6694/12/5/1267/s1, Figure S1: Pearson’s correlation between OvCa stem cell (OCSC) markers. Figure S2: Correlations of OCSC markers with glucose transporters and glycolysis enzymes in OvCa cell lines’ data curated from CCLE. Figure S3: Correlations of OVSC markers with TCA cycle enzymes in OvCa cell lines’ data curated from CCLE. Red indicates positive correlations. Figure S4: Pearson Correlation between the expression of OCSC transcripts and the transcripts of the key enzymes involved in ETC in OvCa cell lines’ data curated from CCLE. Figure S5: Correlations of OCSC markers with fatty acid transporters and lipid metabolism enzymes in OvCa cell lines’ data curated from CCLE. Figure S6: Correlations of OCSC markers with glutamine/glutamine transporters and metabolic enzymes in OvCa cell lines’ data curated from CCLE. Table S1: Pearson Correlation between the expression of CSC transcripts and the transcripts of glucose transporters and the key enzymes involved in glycolysis in OVCAR3 spheroids stem cells curated from GSE28799. Table S2: Pearson Correlation between the expression of CSC transcripts and the transcripts of glucose transporters and the key enzymes involved in glycolysis in undifferentiated spheroids stem cells curated from GSE64999. Table S3: Pearson Correlation between the expression of CSC transcripts and the transcripts of the key TCA enzymes in OVCAR3 stem cells from GSE28799. Table S4: Pearson Correlation between the expression of CSC transcripts and the transcripts of the key TCA enzymes in undifferentiated OCSCs from GSE64999. Table S5: Pearson Correlation between the expression of CSC transcripts and the transcripts of the key enzymes involved in the Electron Transport Chain in OvCa cell lines curated from GSE28799. Table S6: Pearson Correlation between the expression of CSC transcripts and the transcripts of the key enzymes involved in the Electron Transport Chain in OvCa cell lines curated from GSE64999. Table S7: Pearson Correlation between OCSC markers and lipid transporters and key enzymes in lipid metabolism in GSE28799. Table S8: Pearson Correlation between OCSC markers and lipid transporters and key enzymes in lipid metabolism in GSE64999. Table S9: Pearson Correlation between OCSC markers and glutamate transporters and key enzymes of glutamate/glutamine metabolism in GSE28799. Table S10: Pearson Correlation between OCSC markers and glutamate transporters and key enzymes in glutamate/glutamine metabolism in *GSE64999.*
