# Supplementary material for: Metabolic Plasticity in Ovarian Cancer Stem Cells
Source: Cancers (Basel). 2020 May 17;12(5):1267. doi: 10.3390/cancers12051267 (PMC7281273; doi:10.3390/cancers12051267)
Supplement: Supplementary file 1 [file cancers-12-01267-s001.zip › cancers-795049-supplementary.pptx]

## Slide 1
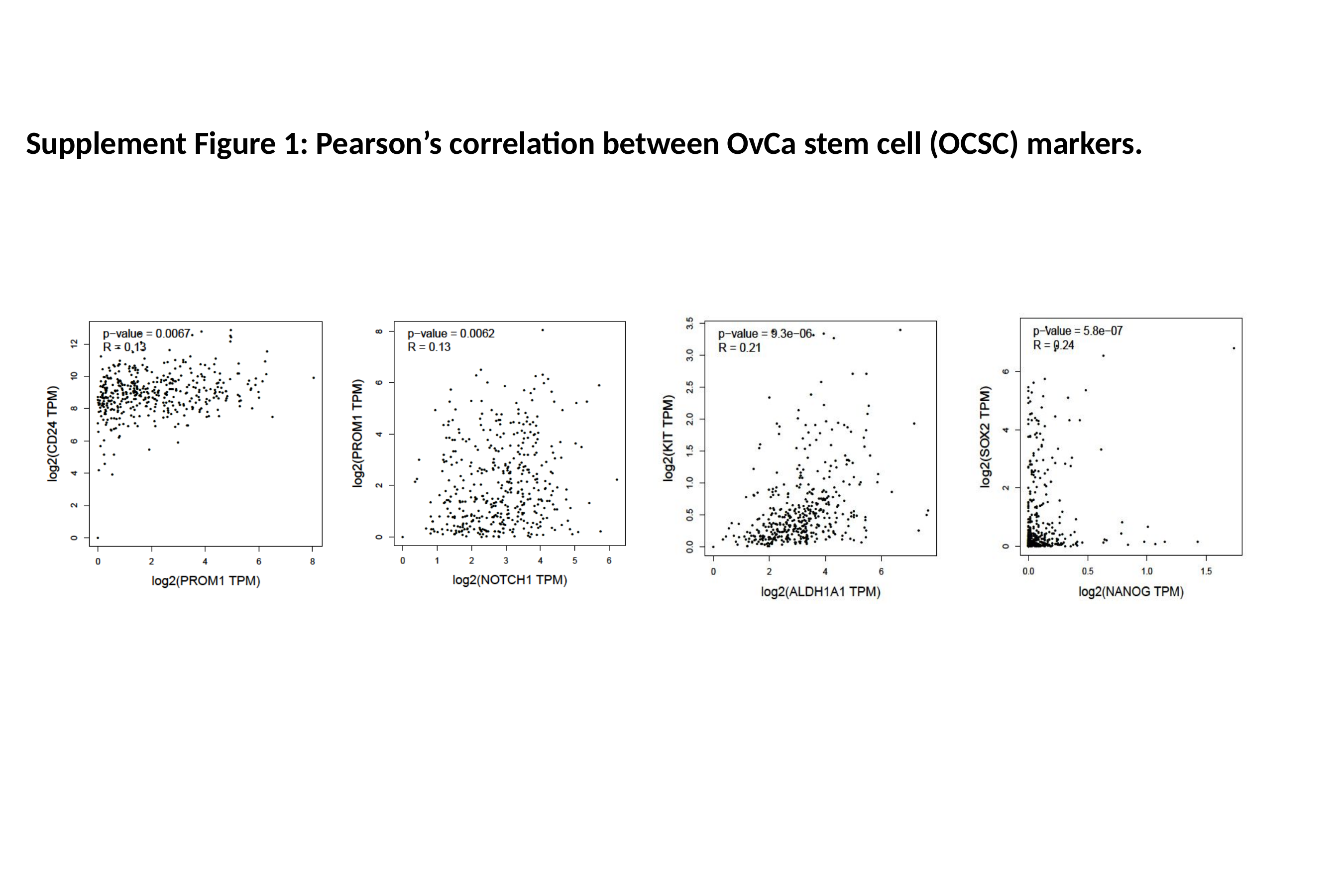

Supplement Figure 1: Pearson’s correlation between OvCa stem cell (OCSC) markers.

## Slide 2
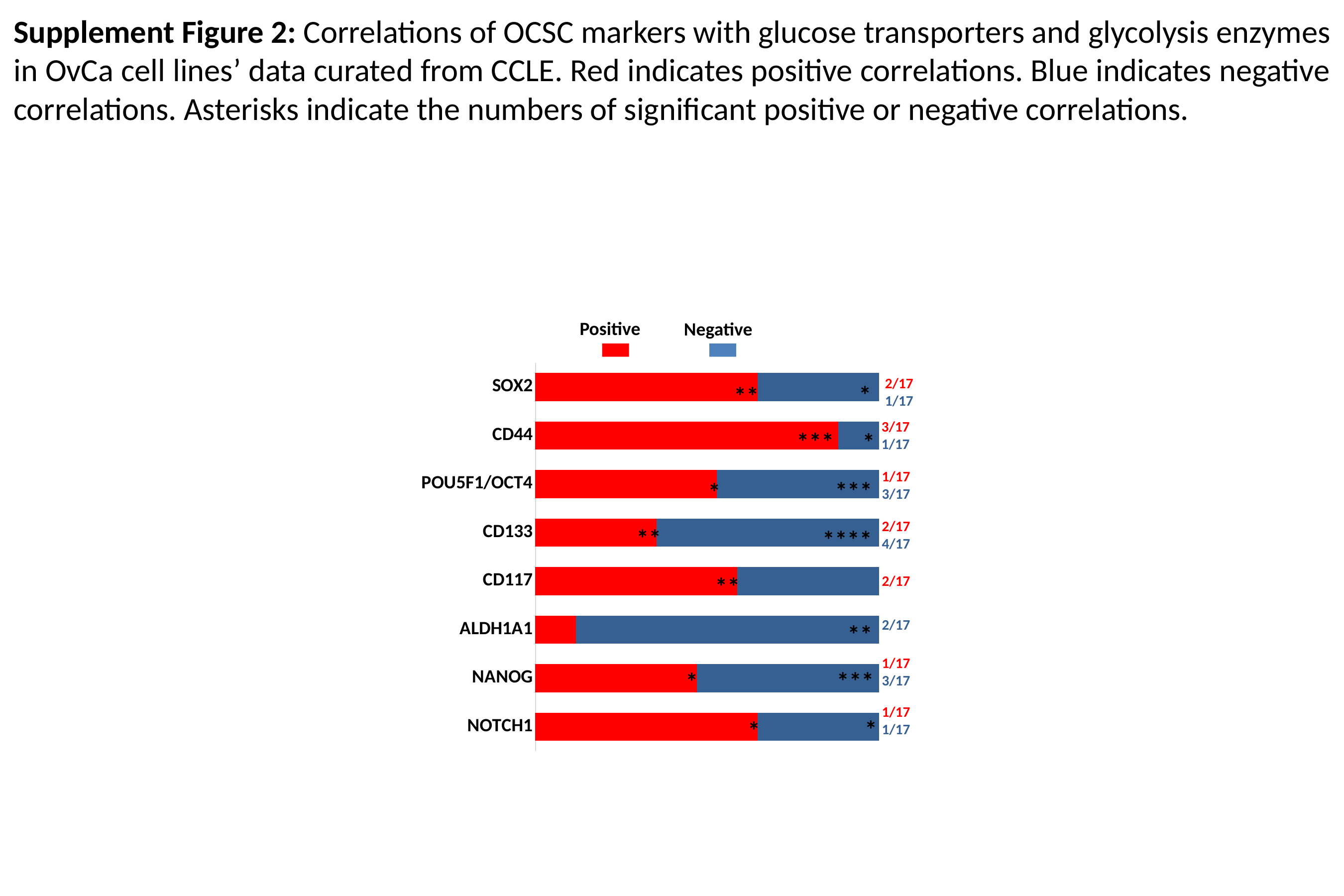

Supplement Figure 2: Correlations of OCSC markers with glucose transporters and glycolysis enzymes in OvCa cell lines’ data curated from CCLE. Red indicates positive correlations. Blue indicates negative correlations. Asterisks indicate the numbers of significant positive or negative correlations.
Positive
Negative
### Chart
| Category | | |
|---|---|---|
| NOTCH1 | 11.0 | 6.0 |
| NANOG | 8.0 | 9.0 |
| ALDH1A1 | 2.0 | 15.0 |
| CD117 | 10.0 | 7.0 |
| CD133 | 6.0 | 11.0 |
| POU5F1/OCT4 | 9.0 | 8.0 |
| CD44 | 15.0 | 2.0 |
| SOX2 | 11.0 | 6.0 |2/17
1/17
*
**
3/17
1/17
***
*
1/17
3/17
***
*
2/17
4/17
**
****
2/17
**
2/17
**
1/17
3/17
***
*
1/17
1/17
*
*

## Slide 3
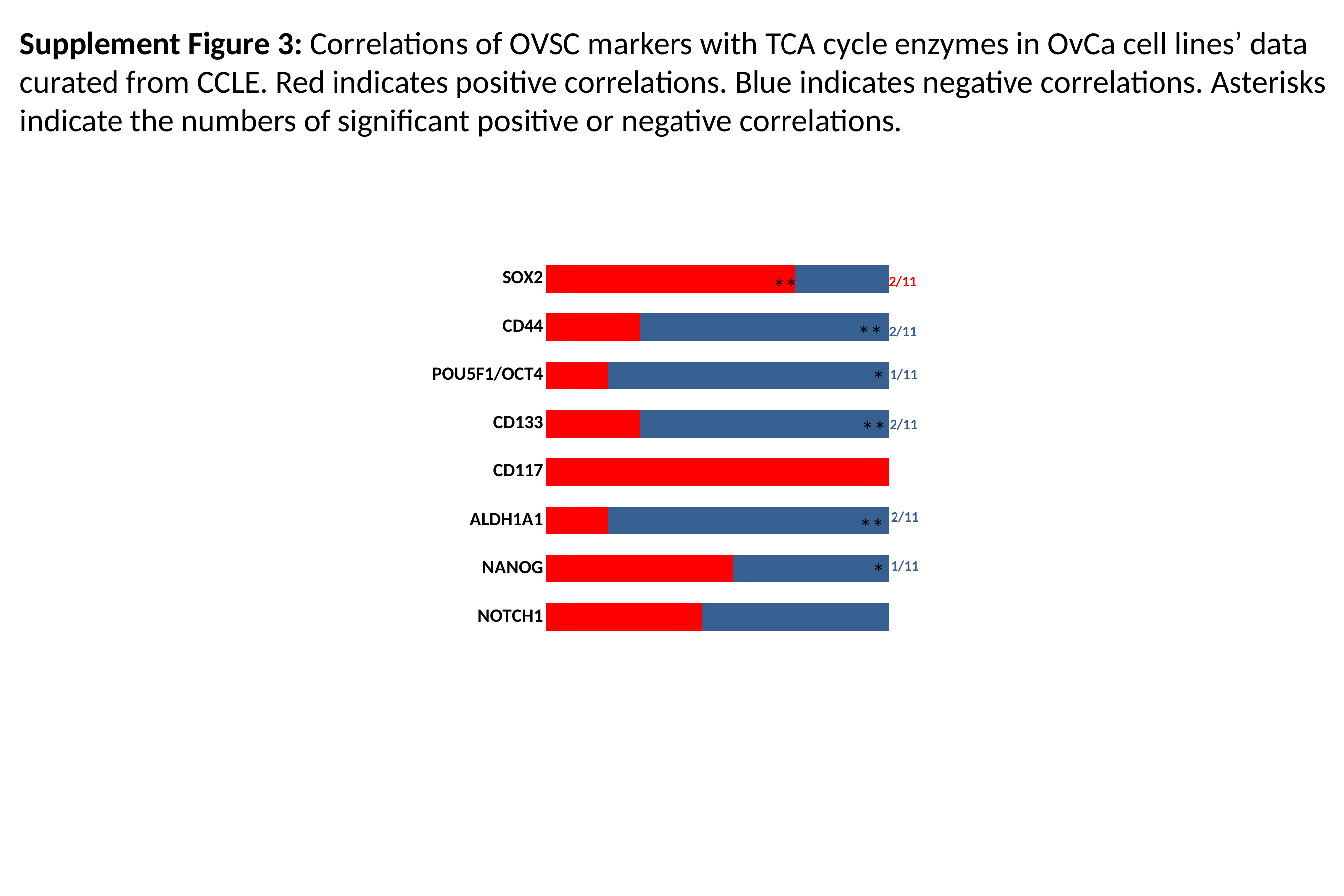

Supplement Figure 3: Correlations of OVSC markers with TCA cycle enzymes in OvCa cell lines’ data curated from CCLE. Red indicates positive correlations. Blue indicates negative correlations. Asterisks indicate the numbers of significant positive or negative correlations.
### Chart
| Category | | |
|---|---|---|
| NOTCH1 | 5.0 | 6.0 |
| NANOG | 6.0 | 5.0 |
| ALDH1A1 | 2.0 | 9.0 |
| CD117 | 11.0 | 0.0 |
| CD133 | 3.0 | 8.0 |
| POU5F1/OCT4 | 2.0 | 9.0 |
| CD44 | 3.0 | 8.0 |
| SOX2 | 8.0 | 3.0 |**
2/11
**
2/11
*
1/11
**
2/11
2/11
**
*
1/11

## Slide 4
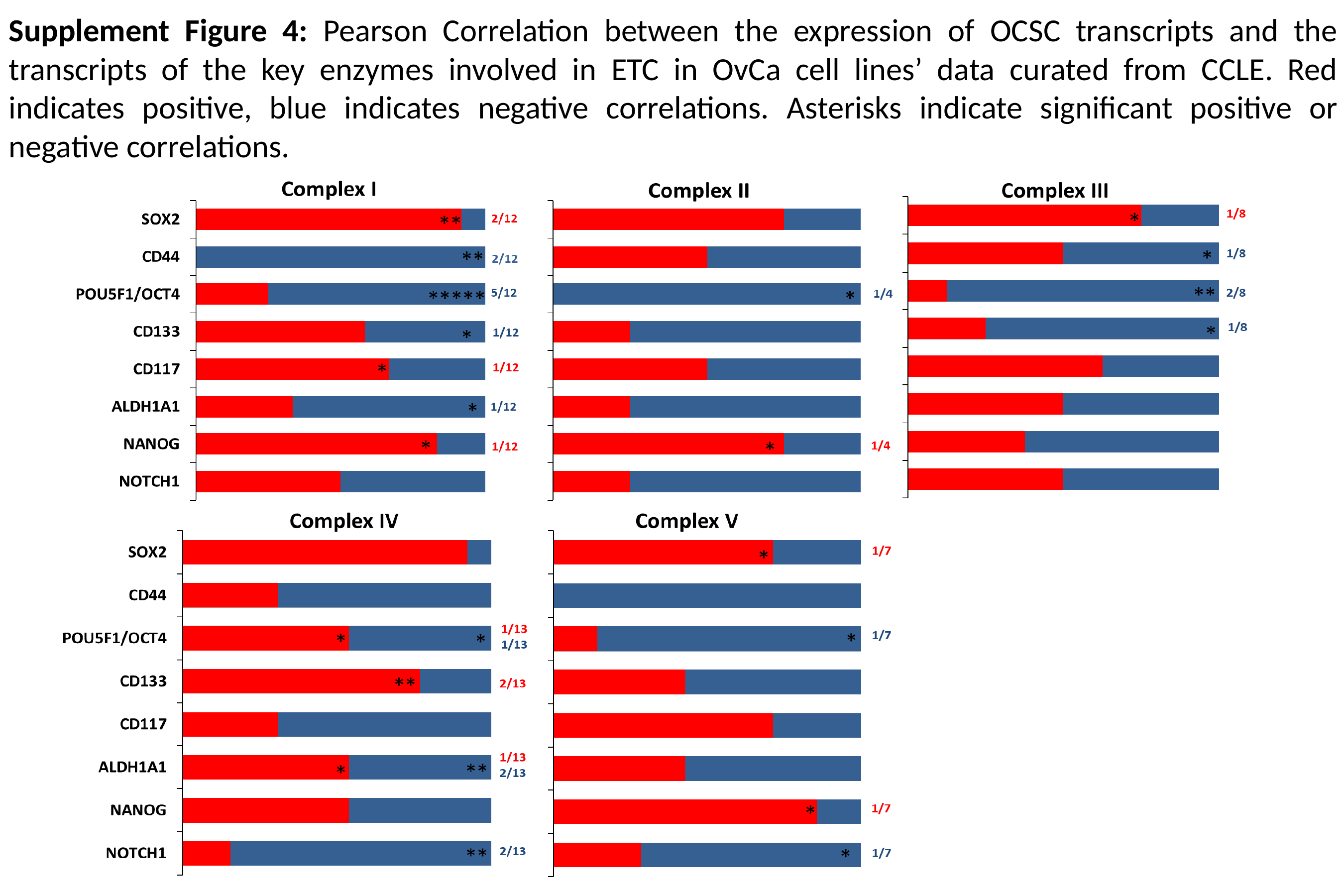

Supplement Figure 4: Pearson Correlation between the expression of OCSC transcripts and the transcripts of the key enzymes involved in ETC in OvCa cell lines’ data curated from CCLE. Red indicates positive, blue indicates negative correlations. Asterisks indicate significant positive or negative correlations.

## Slide 5
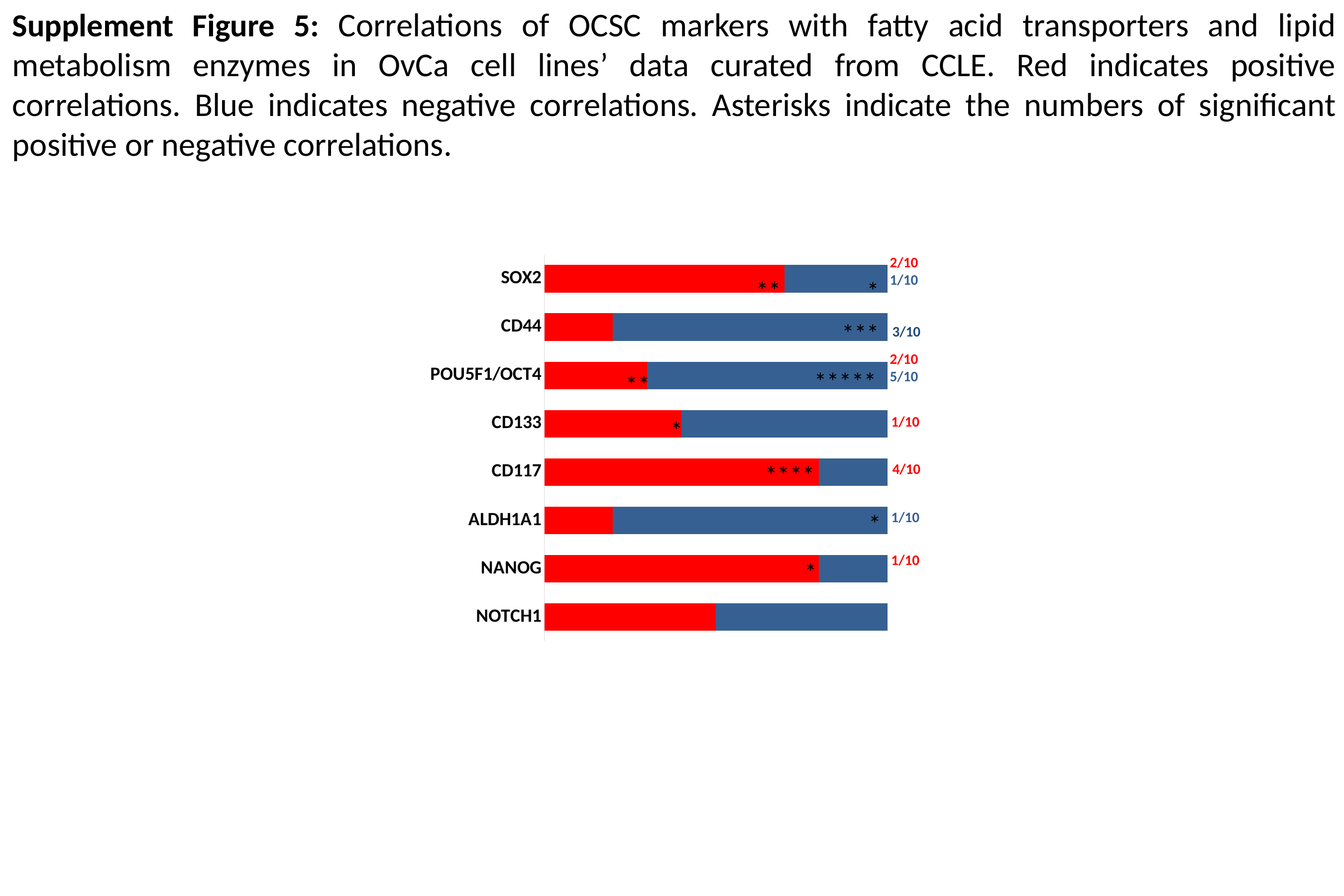

Supplement Figure 5: Correlations of OCSC markers with fatty acid transporters and lipid metabolism enzymes in OvCa cell lines’ data curated from CCLE. Red indicates positive correlations. Blue indicates negative correlations. Asterisks indicate the numbers of significant positive or negative correlations.
### Chart
| Category | | |
|---|---|---|
| NOTCH1 | 5.0 | 5.0 |
| NANOG | 8.0 | 2.0 |
| ALDH1A1 | 2.0 | 8.0 |
| CD117 | 8.0 | 2.0 |
| CD133 | 4.0 | 6.0 |
| POU5F1/OCT4 | 3.0 | 7.0 |
| CD44 | 2.0 | 8.0 |
| SOX2 | 7.0 | 3.0 |2/10
1/10
3/10
***
*****
**
1/10
*
****
4/10
*
1/10
1/10
*
**
*
2/10
5/10

## Slide 6
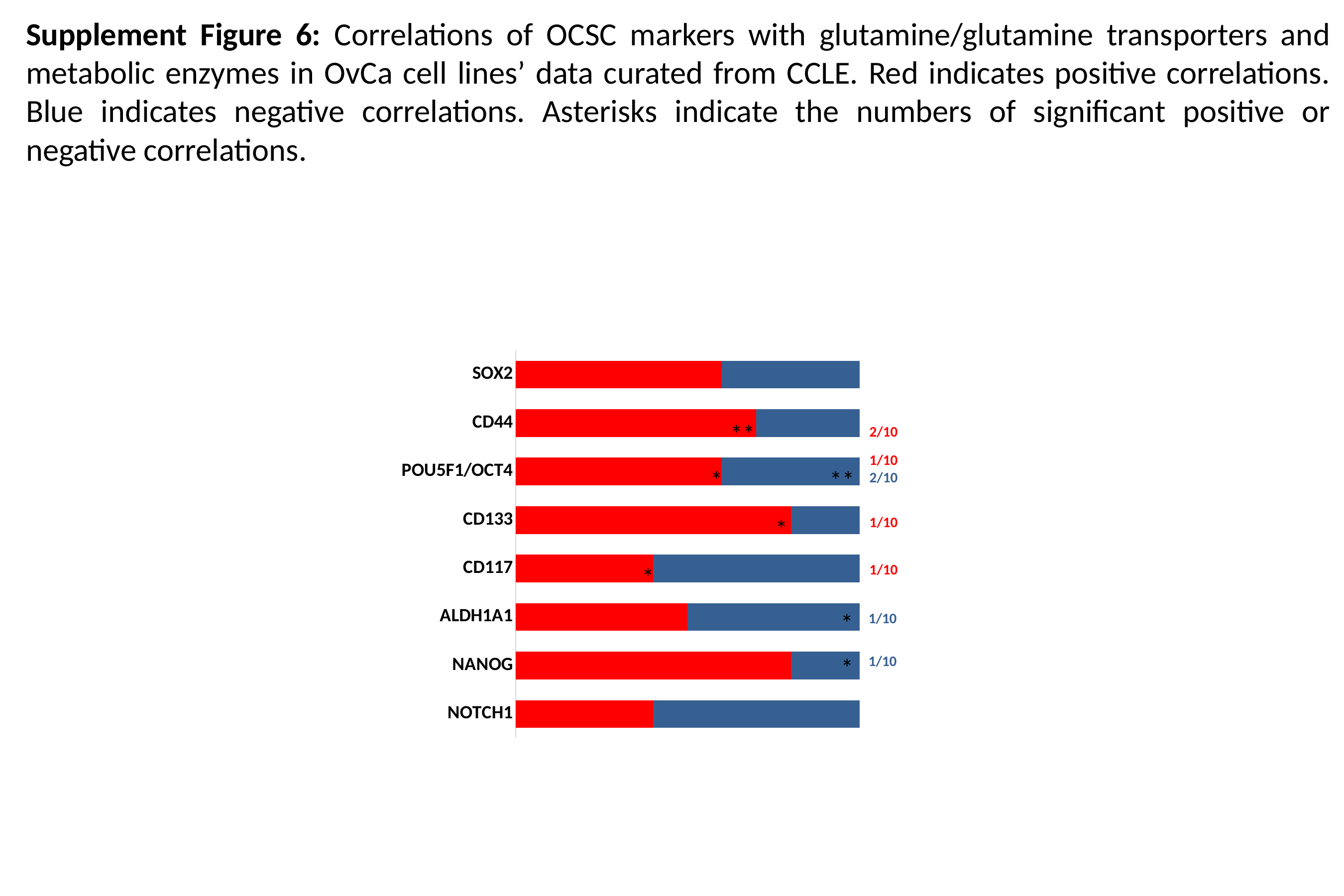

Supplement Figure 6: Correlations of OCSC markers with glutamine/glutamine transporters and metabolic enzymes in OvCa cell lines’ data curated from CCLE. Red indicates positive correlations. Blue indicates negative correlations. Asterisks indicate the numbers of significant positive or negative correlations.
### Chart
| Category | | |
|---|---|---|
| NOTCH1 | 4.0 | 6.0 |
| NANOG | 8.0 | 2.0 |
| ALDH1A1 | 5.0 | 5.0 |
| CD117 | 4.0 | 6.0 |
| CD133 | 8.0 | 2.0 |
| POU5F1/OCT4 | 6.0 | 4.0 |
| CD44 | 7.0 | 3.0 |
| SOX2 | 6.0 | 4.0 |
2/10
**
1/10
2/10
*
**
*
1/10
1/10
*
*
1/10
*
1/10

## Slide 7
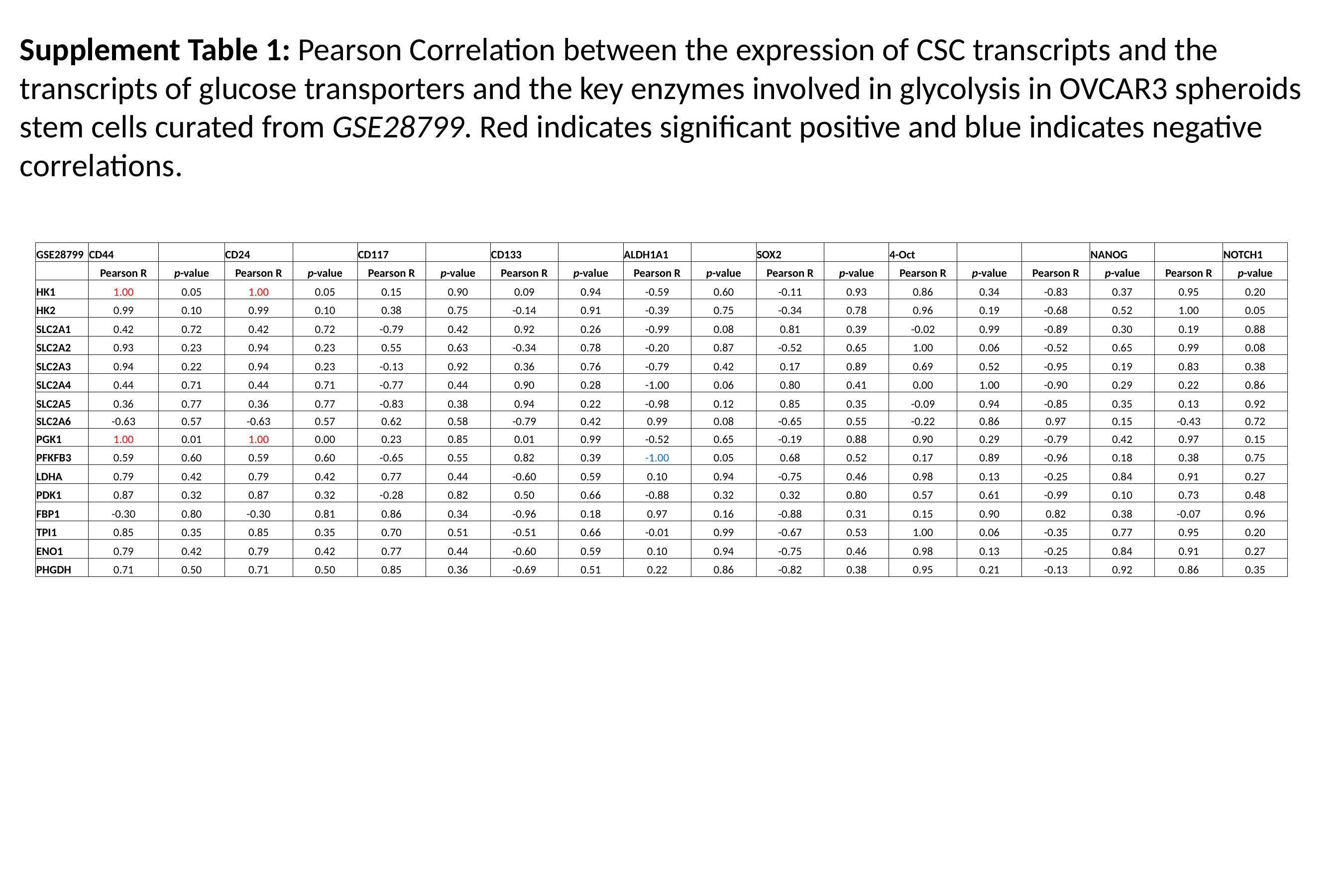

Supplement Table 1: Pearson Correlation between the expression of CSC transcripts and the transcripts of glucose transporters and the key enzymes involved in glycolysis in OVCAR3 spheroids stem cells curated from GSE28799. Red indicates significant positive and blue indicates negative correlations.
| GSE28799 | CD44 | | CD24 | | CD117 | | CD133 | | ALDH1A1 | | SOX2 | | 4-Oct | | | NANOG | | NOTCH1 |
| --- | --- | --- | --- | --- | --- | --- | --- | --- | --- | --- | --- | --- | --- | --- | --- | --- | --- | --- |
| | Pearson R | p-value | Pearson R | p-value | Pearson R | p-value | Pearson R | p-value | Pearson R | p-value | Pearson R | p-value | Pearson R | p-value | Pearson R | p-value | Pearson R | p-value |
| HK1 | 1.00 | 0.05 | 1.00 | 0.05 | 0.15 | 0.90 | 0.09 | 0.94 | -0.59 | 0.60 | -0.11 | 0.93 | 0.86 | 0.34 | -0.83 | 0.37 | 0.95 | 0.20 |
| HK2 | 0.99 | 0.10 | 0.99 | 0.10 | 0.38 | 0.75 | -0.14 | 0.91 | -0.39 | 0.75 | -0.34 | 0.78 | 0.96 | 0.19 | -0.68 | 0.52 | 1.00 | 0.05 |
| SLC2A1 | 0.42 | 0.72 | 0.42 | 0.72 | -0.79 | 0.42 | 0.92 | 0.26 | -0.99 | 0.08 | 0.81 | 0.39 | -0.02 | 0.99 | -0.89 | 0.30 | 0.19 | 0.88 |
| SLC2A2 | 0.93 | 0.23 | 0.94 | 0.23 | 0.55 | 0.63 | -0.34 | 0.78 | -0.20 | 0.87 | -0.52 | 0.65 | 1.00 | 0.06 | -0.52 | 0.65 | 0.99 | 0.08 |
| SLC2A3 | 0.94 | 0.22 | 0.94 | 0.23 | -0.13 | 0.92 | 0.36 | 0.76 | -0.79 | 0.42 | 0.17 | 0.89 | 0.69 | 0.52 | -0.95 | 0.19 | 0.83 | 0.38 |
| SLC2A4 | 0.44 | 0.71 | 0.44 | 0.71 | -0.77 | 0.44 | 0.90 | 0.28 | -1.00 | 0.06 | 0.80 | 0.41 | 0.00 | 1.00 | -0.90 | 0.29 | 0.22 | 0.86 |
| SLC2A5 | 0.36 | 0.77 | 0.36 | 0.77 | -0.83 | 0.38 | 0.94 | 0.22 | -0.98 | 0.12 | 0.85 | 0.35 | -0.09 | 0.94 | -0.85 | 0.35 | 0.13 | 0.92 |
| SLC2A6 | -0.63 | 0.57 | -0.63 | 0.57 | 0.62 | 0.58 | -0.79 | 0.42 | 0.99 | 0.08 | -0.65 | 0.55 | -0.22 | 0.86 | 0.97 | 0.15 | -0.43 | 0.72 |
| PGK1 | 1.00 | 0.01 | 1.00 | 0.00 | 0.23 | 0.85 | 0.01 | 0.99 | -0.52 | 0.65 | -0.19 | 0.88 | 0.90 | 0.29 | -0.79 | 0.42 | 0.97 | 0.15 |
| PFKFB3 | 0.59 | 0.60 | 0.59 | 0.60 | -0.65 | 0.55 | 0.82 | 0.39 | -1.00 | 0.05 | 0.68 | 0.52 | 0.17 | 0.89 | -0.96 | 0.18 | 0.38 | 0.75 |
| LDHA | 0.79 | 0.42 | 0.79 | 0.42 | 0.77 | 0.44 | -0.60 | 0.59 | 0.10 | 0.94 | -0.75 | 0.46 | 0.98 | 0.13 | -0.25 | 0.84 | 0.91 | 0.27 |
| PDK1 | 0.87 | 0.32 | 0.87 | 0.32 | -0.28 | 0.82 | 0.50 | 0.66 | -0.88 | 0.32 | 0.32 | 0.80 | 0.57 | 0.61 | -0.99 | 0.10 | 0.73 | 0.48 |
| FBP1 | -0.30 | 0.80 | -0.30 | 0.81 | 0.86 | 0.34 | -0.96 | 0.18 | 0.97 | 0.16 | -0.88 | 0.31 | 0.15 | 0.90 | 0.82 | 0.38 | -0.07 | 0.96 |
| TPI1 | 0.85 | 0.35 | 0.85 | 0.35 | 0.70 | 0.51 | -0.51 | 0.66 | -0.01 | 0.99 | -0.67 | 0.53 | 1.00 | 0.06 | -0.35 | 0.77 | 0.95 | 0.20 |
| ENO1 | 0.79 | 0.42 | 0.79 | 0.42 | 0.77 | 0.44 | -0.60 | 0.59 | 0.10 | 0.94 | -0.75 | 0.46 | 0.98 | 0.13 | -0.25 | 0.84 | 0.91 | 0.27 |
| PHGDH | 0.71 | 0.50 | 0.71 | 0.50 | 0.85 | 0.36 | -0.69 | 0.51 | 0.22 | 0.86 | -0.82 | 0.38 | 0.95 | 0.21 | -0.13 | 0.92 | 0.86 | 0.35 |

## Slide 8
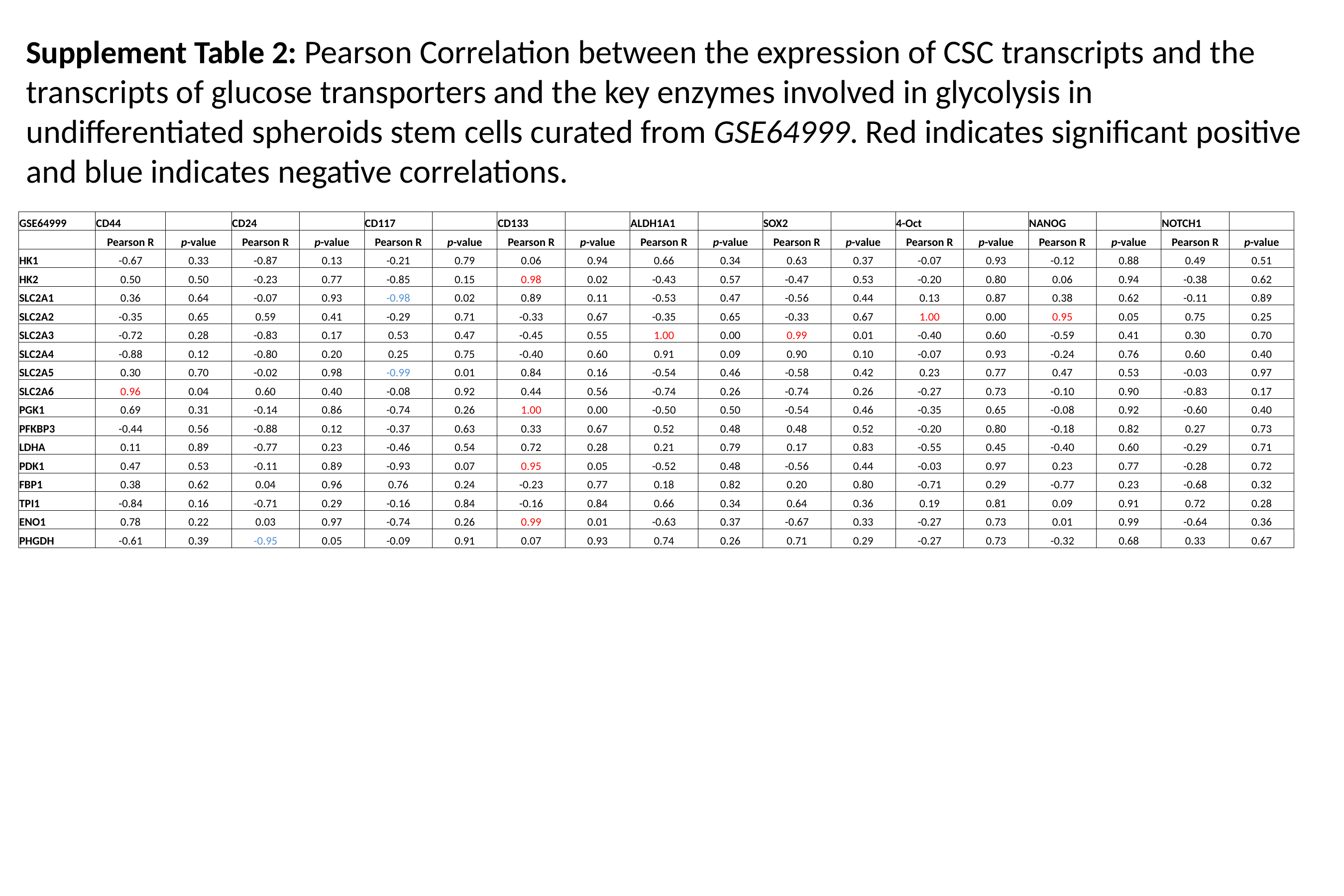

Supplement Table 2: Pearson Correlation between the expression of CSC transcripts and the transcripts of glucose transporters and the key enzymes involved in glycolysis in undifferentiated spheroids stem cells curated from GSE64999. Red indicates significant positive and blue indicates negative correlations.
| GSE64999 | CD44 | | CD24 | | CD117 | | CD133 | | ALDH1A1 | | SOX2 | | 4-Oct | | NANOG | | NOTCH1 | |
| --- | --- | --- | --- | --- | --- | --- | --- | --- | --- | --- | --- | --- | --- | --- | --- | --- | --- | --- |
| | Pearson R | p-value | Pearson R | p-value | Pearson R | p-value | Pearson R | p-value | Pearson R | p-value | Pearson R | p-value | Pearson R | p-value | Pearson R | p-value | Pearson R | p-value |
| HK1 | -0.67 | 0.33 | -0.87 | 0.13 | -0.21 | 0.79 | 0.06 | 0.94 | 0.66 | 0.34 | 0.63 | 0.37 | -0.07 | 0.93 | -0.12 | 0.88 | 0.49 | 0.51 |
| HK2 | 0.50 | 0.50 | -0.23 | 0.77 | -0.85 | 0.15 | 0.98 | 0.02 | -0.43 | 0.57 | -0.47 | 0.53 | -0.20 | 0.80 | 0.06 | 0.94 | -0.38 | 0.62 |
| SLC2A1 | 0.36 | 0.64 | -0.07 | 0.93 | -0.98 | 0.02 | 0.89 | 0.11 | -0.53 | 0.47 | -0.56 | 0.44 | 0.13 | 0.87 | 0.38 | 0.62 | -0.11 | 0.89 |
| SLC2A2 | -0.35 | 0.65 | 0.59 | 0.41 | -0.29 | 0.71 | -0.33 | 0.67 | -0.35 | 0.65 | -0.33 | 0.67 | 1.00 | 0.00 | 0.95 | 0.05 | 0.75 | 0.25 |
| SLC2A3 | -0.72 | 0.28 | -0.83 | 0.17 | 0.53 | 0.47 | -0.45 | 0.55 | 1.00 | 0.00 | 0.99 | 0.01 | -0.40 | 0.60 | -0.59 | 0.41 | 0.30 | 0.70 |
| SLC2A4 | -0.88 | 0.12 | -0.80 | 0.20 | 0.25 | 0.75 | -0.40 | 0.60 | 0.91 | 0.09 | 0.90 | 0.10 | -0.07 | 0.93 | -0.24 | 0.76 | 0.60 | 0.40 |
| SLC2A5 | 0.30 | 0.70 | -0.02 | 0.98 | -0.99 | 0.01 | 0.84 | 0.16 | -0.54 | 0.46 | -0.58 | 0.42 | 0.23 | 0.77 | 0.47 | 0.53 | -0.03 | 0.97 |
| SLC2A6 | 0.96 | 0.04 | 0.60 | 0.40 | -0.08 | 0.92 | 0.44 | 0.56 | -0.74 | 0.26 | -0.74 | 0.26 | -0.27 | 0.73 | -0.10 | 0.90 | -0.83 | 0.17 |
| PGK1 | 0.69 | 0.31 | -0.14 | 0.86 | -0.74 | 0.26 | 1.00 | 0.00 | -0.50 | 0.50 | -0.54 | 0.46 | -0.35 | 0.65 | -0.08 | 0.92 | -0.60 | 0.40 |
| PFKBP3 | -0.44 | 0.56 | -0.88 | 0.12 | -0.37 | 0.63 | 0.33 | 0.67 | 0.52 | 0.48 | 0.48 | 0.52 | -0.20 | 0.80 | -0.18 | 0.82 | 0.27 | 0.73 |
| LDHA | 0.11 | 0.89 | -0.77 | 0.23 | -0.46 | 0.54 | 0.72 | 0.28 | 0.21 | 0.79 | 0.17 | 0.83 | -0.55 | 0.45 | -0.40 | 0.60 | -0.29 | 0.71 |
| PDK1 | 0.47 | 0.53 | -0.11 | 0.89 | -0.93 | 0.07 | 0.95 | 0.05 | -0.52 | 0.48 | -0.56 | 0.44 | -0.03 | 0.97 | 0.23 | 0.77 | -0.28 | 0.72 |
| FBP1 | 0.38 | 0.62 | 0.04 | 0.96 | 0.76 | 0.24 | -0.23 | 0.77 | 0.18 | 0.82 | 0.20 | 0.80 | -0.71 | 0.29 | -0.77 | 0.23 | -0.68 | 0.32 |
| TPI1 | -0.84 | 0.16 | -0.71 | 0.29 | -0.16 | 0.84 | -0.16 | 0.84 | 0.66 | 0.34 | 0.64 | 0.36 | 0.19 | 0.81 | 0.09 | 0.91 | 0.72 | 0.28 |
| ENO1 | 0.78 | 0.22 | 0.03 | 0.97 | -0.74 | 0.26 | 0.99 | 0.01 | -0.63 | 0.37 | -0.67 | 0.33 | -0.27 | 0.73 | 0.01 | 0.99 | -0.64 | 0.36 |
| PHGDH | -0.61 | 0.39 | -0.95 | 0.05 | -0.09 | 0.91 | 0.07 | 0.93 | 0.74 | 0.26 | 0.71 | 0.29 | -0.27 | 0.73 | -0.32 | 0.68 | 0.33 | 0.67 |

## Slide 9
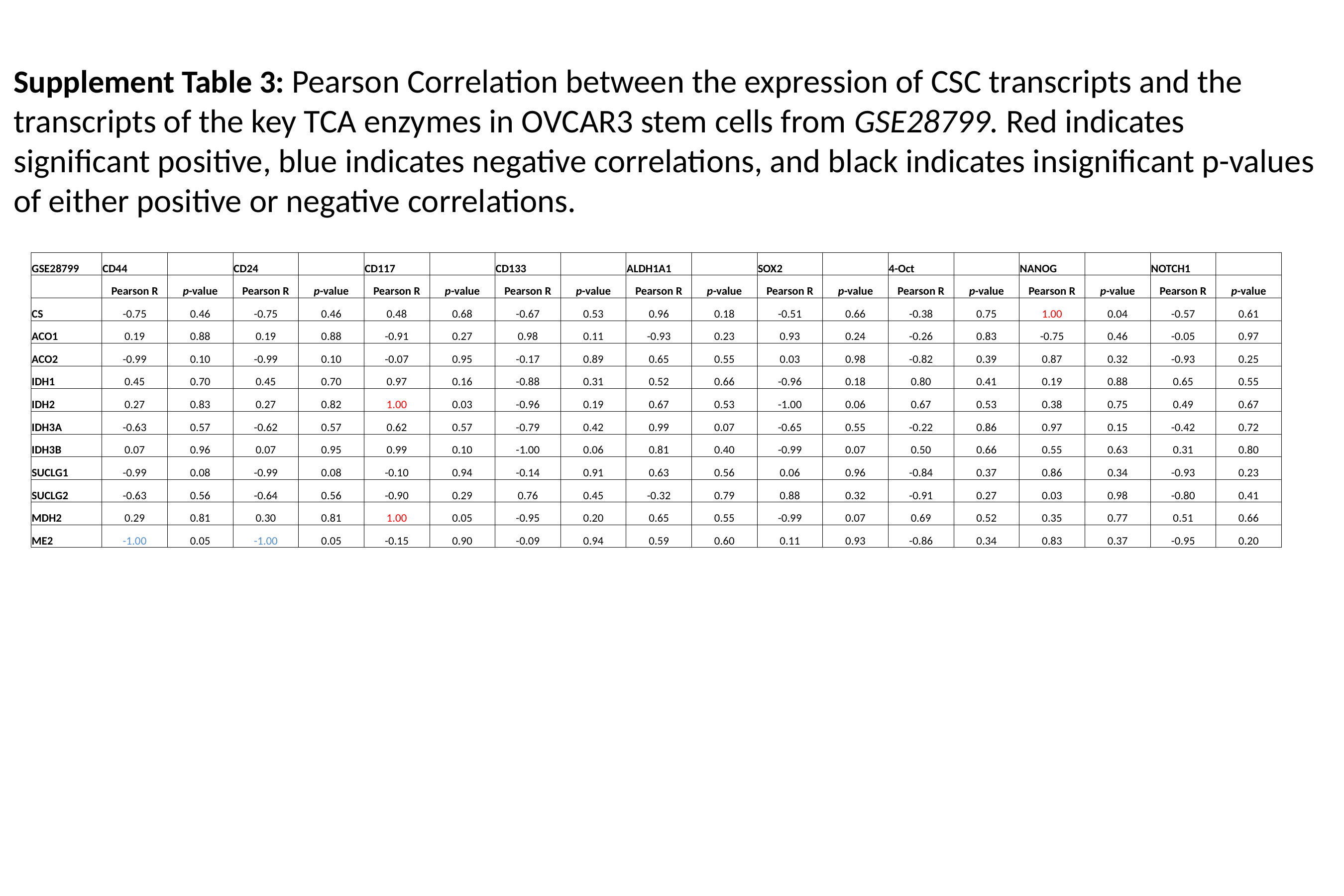

Supplement Table 3: Pearson Correlation between the expression of CSC transcripts and the transcripts of the key TCA enzymes in OVCAR3 stem cells from GSE28799. Red indicates significant positive, blue indicates negative correlations, and black indicates insignificant p-values of either positive or negative correlations.
| GSE28799 | CD44 | | CD24 | | CD117 | | CD133 | | ALDH1A1 | | SOX2 | | 4-Oct | | NANOG | | NOTCH1 | |
| --- | --- | --- | --- | --- | --- | --- | --- | --- | --- | --- | --- | --- | --- | --- | --- | --- | --- | --- |
| | Pearson R | p-value | Pearson R | p-value | Pearson R | p-value | Pearson R | p-value | Pearson R | p-value | Pearson R | p-value | Pearson R | p-value | Pearson R | p-value | Pearson R | p-value |
| CS | -0.75 | 0.46 | -0.75 | 0.46 | 0.48 | 0.68 | -0.67 | 0.53 | 0.96 | 0.18 | -0.51 | 0.66 | -0.38 | 0.75 | 1.00 | 0.04 | -0.57 | 0.61 |
| ACO1 | 0.19 | 0.88 | 0.19 | 0.88 | -0.91 | 0.27 | 0.98 | 0.11 | -0.93 | 0.23 | 0.93 | 0.24 | -0.26 | 0.83 | -0.75 | 0.46 | -0.05 | 0.97 |
| ACO2 | -0.99 | 0.10 | -0.99 | 0.10 | -0.07 | 0.95 | -0.17 | 0.89 | 0.65 | 0.55 | 0.03 | 0.98 | -0.82 | 0.39 | 0.87 | 0.32 | -0.93 | 0.25 |
| IDH1 | 0.45 | 0.70 | 0.45 | 0.70 | 0.97 | 0.16 | -0.88 | 0.31 | 0.52 | 0.66 | -0.96 | 0.18 | 0.80 | 0.41 | 0.19 | 0.88 | 0.65 | 0.55 |
| IDH2 | 0.27 | 0.83 | 0.27 | 0.82 | 1.00 | 0.03 | -0.96 | 0.19 | 0.67 | 0.53 | -1.00 | 0.06 | 0.67 | 0.53 | 0.38 | 0.75 | 0.49 | 0.67 |
| IDH3A | -0.63 | 0.57 | -0.62 | 0.57 | 0.62 | 0.57 | -0.79 | 0.42 | 0.99 | 0.07 | -0.65 | 0.55 | -0.22 | 0.86 | 0.97 | 0.15 | -0.42 | 0.72 |
| IDH3B | 0.07 | 0.96 | 0.07 | 0.95 | 0.99 | 0.10 | -1.00 | 0.06 | 0.81 | 0.40 | -0.99 | 0.07 | 0.50 | 0.66 | 0.55 | 0.63 | 0.31 | 0.80 |
| SUCLG1 | -0.99 | 0.08 | -0.99 | 0.08 | -0.10 | 0.94 | -0.14 | 0.91 | 0.63 | 0.56 | 0.06 | 0.96 | -0.84 | 0.37 | 0.86 | 0.34 | -0.93 | 0.23 |
| SUCLG2 | -0.63 | 0.56 | -0.64 | 0.56 | -0.90 | 0.29 | 0.76 | 0.45 | -0.32 | 0.79 | 0.88 | 0.32 | -0.91 | 0.27 | 0.03 | 0.98 | -0.80 | 0.41 |
| MDH2 | 0.29 | 0.81 | 0.30 | 0.81 | 1.00 | 0.05 | -0.95 | 0.20 | 0.65 | 0.55 | -0.99 | 0.07 | 0.69 | 0.52 | 0.35 | 0.77 | 0.51 | 0.66 |
| ME2 | -1.00 | 0.05 | -1.00 | 0.05 | -0.15 | 0.90 | -0.09 | 0.94 | 0.59 | 0.60 | 0.11 | 0.93 | -0.86 | 0.34 | 0.83 | 0.37 | -0.95 | 0.20 |

## Slide 10
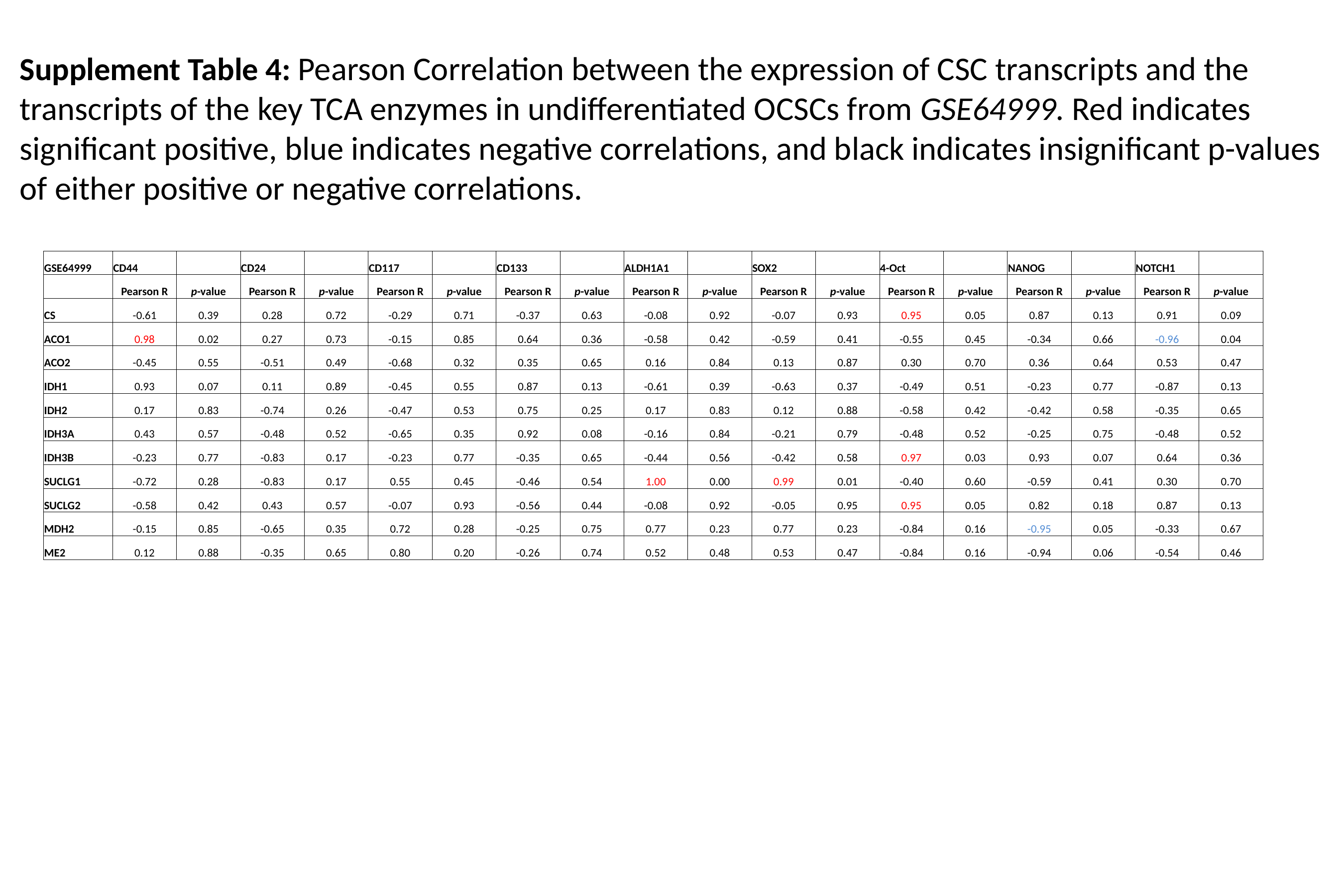

Supplement Table 4: Pearson Correlation between the expression of CSC transcripts and the transcripts of the key TCA enzymes in undifferentiated OCSCs from GSE64999. Red indicates significant positive, blue indicates negative correlations, and black indicates insignificant p-values of either positive or negative correlations.
| GSE64999 | CD44 | | CD24 | | CD117 | | CD133 | | ALDH1A1 | | SOX2 | | 4-Oct | | NANOG | | NOTCH1 | |
| --- | --- | --- | --- | --- | --- | --- | --- | --- | --- | --- | --- | --- | --- | --- | --- | --- | --- | --- |
| | Pearson R | p-value | Pearson R | p-value | Pearson R | p-value | Pearson R | p-value | Pearson R | p-value | Pearson R | p-value | Pearson R | p-value | Pearson R | p-value | Pearson R | p-value |
| CS | -0.61 | 0.39 | 0.28 | 0.72 | -0.29 | 0.71 | -0.37 | 0.63 | -0.08 | 0.92 | -0.07 | 0.93 | 0.95 | 0.05 | 0.87 | 0.13 | 0.91 | 0.09 |
| ACO1 | 0.98 | 0.02 | 0.27 | 0.73 | -0.15 | 0.85 | 0.64 | 0.36 | -0.58 | 0.42 | -0.59 | 0.41 | -0.55 | 0.45 | -0.34 | 0.66 | -0.96 | 0.04 |
| ACO2 | -0.45 | 0.55 | -0.51 | 0.49 | -0.68 | 0.32 | 0.35 | 0.65 | 0.16 | 0.84 | 0.13 | 0.87 | 0.30 | 0.70 | 0.36 | 0.64 | 0.53 | 0.47 |
| IDH1 | 0.93 | 0.07 | 0.11 | 0.89 | -0.45 | 0.55 | 0.87 | 0.13 | -0.61 | 0.39 | -0.63 | 0.37 | -0.49 | 0.51 | -0.23 | 0.77 | -0.87 | 0.13 |
| IDH2 | 0.17 | 0.83 | -0.74 | 0.26 | -0.47 | 0.53 | 0.75 | 0.25 | 0.17 | 0.83 | 0.12 | 0.88 | -0.58 | 0.42 | -0.42 | 0.58 | -0.35 | 0.65 |
| IDH3A | 0.43 | 0.57 | -0.48 | 0.52 | -0.65 | 0.35 | 0.92 | 0.08 | -0.16 | 0.84 | -0.21 | 0.79 | -0.48 | 0.52 | -0.25 | 0.75 | -0.48 | 0.52 |
| IDH3B | -0.23 | 0.77 | -0.83 | 0.17 | -0.23 | 0.77 | -0.35 | 0.65 | -0.44 | 0.56 | -0.42 | 0.58 | 0.97 | 0.03 | 0.93 | 0.07 | 0.64 | 0.36 |
| SUCLG1 | -0.72 | 0.28 | -0.83 | 0.17 | 0.55 | 0.45 | -0.46 | 0.54 | 1.00 | 0.00 | 0.99 | 0.01 | -0.40 | 0.60 | -0.59 | 0.41 | 0.30 | 0.70 |
| SUCLG2 | -0.58 | 0.42 | 0.43 | 0.57 | -0.07 | 0.93 | -0.56 | 0.44 | -0.08 | 0.92 | -0.05 | 0.95 | 0.95 | 0.05 | 0.82 | 0.18 | 0.87 | 0.13 |
| MDH2 | -0.15 | 0.85 | -0.65 | 0.35 | 0.72 | 0.28 | -0.25 | 0.75 | 0.77 | 0.23 | 0.77 | 0.23 | -0.84 | 0.16 | -0.95 | 0.05 | -0.33 | 0.67 |
| ME2 | 0.12 | 0.88 | -0.35 | 0.65 | 0.80 | 0.20 | -0.26 | 0.74 | 0.52 | 0.48 | 0.53 | 0.47 | -0.84 | 0.16 | -0.94 | 0.06 | -0.54 | 0.46 |

## Slide 11
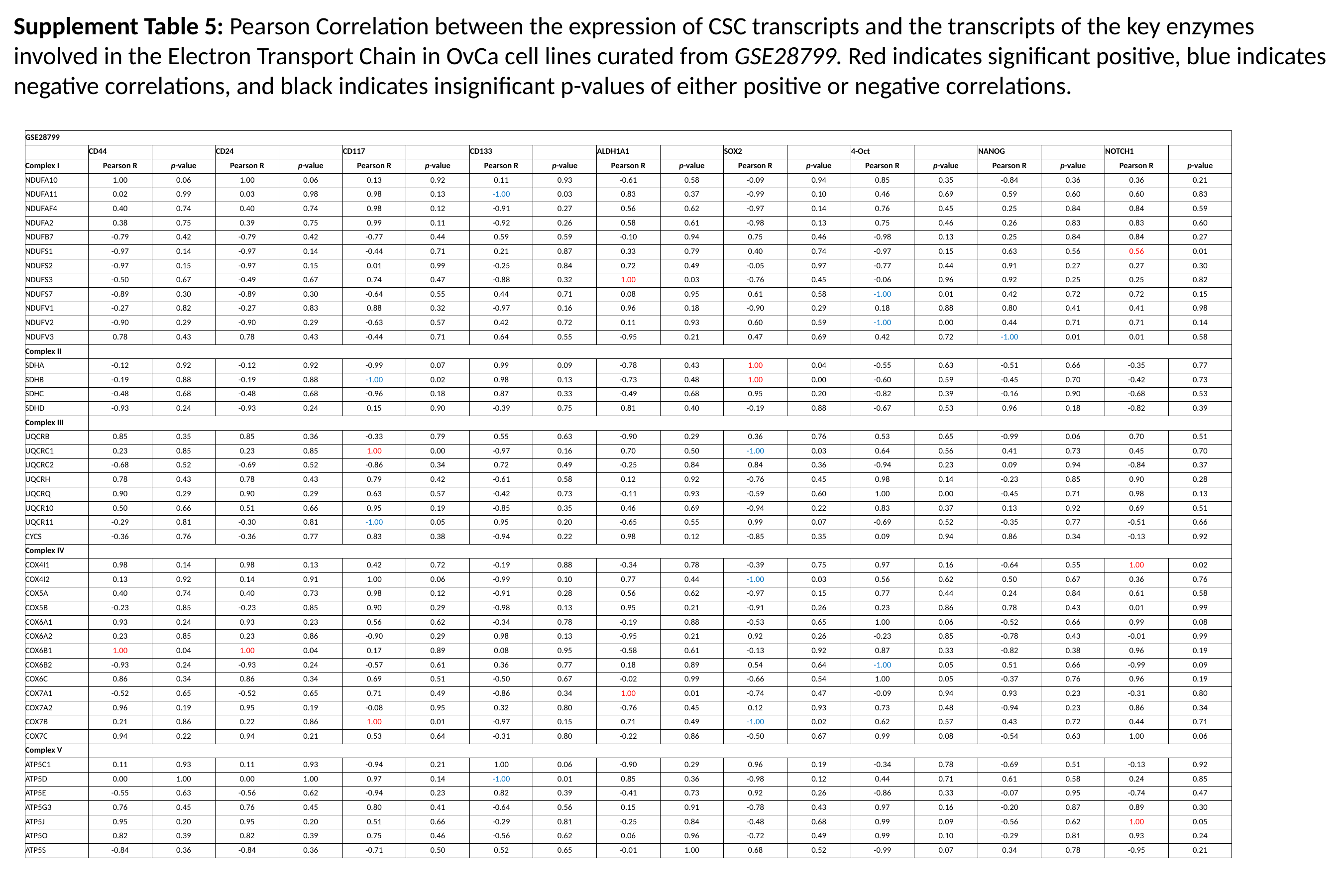

Supplement Table 5: Pearson Correlation between the expression of CSC transcripts and the transcripts of the key enzymes involved in the Electron Transport Chain in OvCa cell lines curated from GSE28799. Red indicates significant positive, blue indicates negative correlations, and black indicates insignificant p-values of either positive or negative correlations.
| GSE28799 | | | | | | | | | | | | | | | | | | |
| --- | --- | --- | --- | --- | --- | --- | --- | --- | --- | --- | --- | --- | --- | --- | --- | --- | --- | --- |
| | CD44 | | CD24 | | CD117 | | CD133 | | ALDH1A1 | | SOX2 | | 4-Oct | | NANOG | | NOTCH1 | |
| Complex I | Pearson R | p-value | Pearson R | p-value | Pearson R | p-value | Pearson R | p-value | Pearson R | p-value | Pearson R | p-value | Pearson R | p-value | Pearson R | p-value | Pearson R | p-value |
| NDUFA10 | 1.00 | 0.06 | 1.00 | 0.06 | 0.13 | 0.92 | 0.11 | 0.93 | -0.61 | 0.58 | -0.09 | 0.94 | 0.85 | 0.35 | -0.84 | 0.36 | 0.36 | 0.21 |
| NDUFA11 | 0.02 | 0.99 | 0.03 | 0.98 | 0.98 | 0.13 | -1.00 | 0.03 | 0.83 | 0.37 | -0.99 | 0.10 | 0.46 | 0.69 | 0.59 | 0.60 | 0.60 | 0.83 |
| NDUFAF4 | 0.40 | 0.74 | 0.40 | 0.74 | 0.98 | 0.12 | -0.91 | 0.27 | 0.56 | 0.62 | -0.97 | 0.14 | 0.76 | 0.45 | 0.25 | 0.84 | 0.84 | 0.59 |
| NDUFA2 | 0.38 | 0.75 | 0.39 | 0.75 | 0.99 | 0.11 | -0.92 | 0.26 | 0.58 | 0.61 | -0.98 | 0.13 | 0.75 | 0.46 | 0.26 | 0.83 | 0.83 | 0.60 |
| NDUFB7 | -0.79 | 0.42 | -0.79 | 0.42 | -0.77 | 0.44 | 0.59 | 0.59 | -0.10 | 0.94 | 0.75 | 0.46 | -0.98 | 0.13 | 0.25 | 0.84 | 0.84 | 0.27 |
| NDUFS1 | -0.97 | 0.14 | -0.97 | 0.14 | -0.44 | 0.71 | 0.21 | 0.87 | 0.33 | 0.79 | 0.40 | 0.74 | -0.97 | 0.15 | 0.63 | 0.56 | 0.56 | 0.01 |
| NDUFS2 | -0.97 | 0.15 | -0.97 | 0.15 | 0.01 | 0.99 | -0.25 | 0.84 | 0.72 | 0.49 | -0.05 | 0.97 | -0.77 | 0.44 | 0.91 | 0.27 | 0.27 | 0.30 |
| NDUFS3 | -0.50 | 0.67 | -0.49 | 0.67 | 0.74 | 0.47 | -0.88 | 0.32 | 1.00 | 0.03 | -0.76 | 0.45 | -0.06 | 0.96 | 0.92 | 0.25 | 0.25 | 0.82 |
| NDUFS7 | -0.89 | 0.30 | -0.89 | 0.30 | -0.64 | 0.55 | 0.44 | 0.71 | 0.08 | 0.95 | 0.61 | 0.58 | -1.00 | 0.01 | 0.42 | 0.72 | 0.72 | 0.15 |
| NDUFV1 | -0.27 | 0.82 | -0.27 | 0.83 | 0.88 | 0.32 | -0.97 | 0.16 | 0.96 | 0.18 | -0.90 | 0.29 | 0.18 | 0.88 | 0.80 | 0.41 | 0.41 | 0.98 |
| NDUFV2 | -0.90 | 0.29 | -0.90 | 0.29 | -0.63 | 0.57 | 0.42 | 0.72 | 0.11 | 0.93 | 0.60 | 0.59 | -1.00 | 0.00 | 0.44 | 0.71 | 0.71 | 0.14 |
| NDUFV3 | 0.78 | 0.43 | 0.78 | 0.43 | -0.44 | 0.71 | 0.64 | 0.55 | -0.95 | 0.21 | 0.47 | 0.69 | 0.42 | 0.72 | -1.00 | 0.01 | 0.01 | 0.58 |
| Complex II | | | | | | | | | | | | | | | | | | |
| SDHA | -0.12 | 0.92 | -0.12 | 0.92 | -0.99 | 0.07 | 0.99 | 0.09 | -0.78 | 0.43 | 1.00 | 0.04 | -0.55 | 0.63 | -0.51 | 0.66 | -0.35 | 0.77 |
| SDHB | -0.19 | 0.88 | -0.19 | 0.88 | -1.00 | 0.02 | 0.98 | 0.13 | -0.73 | 0.48 | 1.00 | 0.00 | -0.60 | 0.59 | -0.45 | 0.70 | -0.42 | 0.73 |
| SDHC | -0.48 | 0.68 | -0.48 | 0.68 | -0.96 | 0.18 | 0.87 | 0.33 | -0.49 | 0.68 | 0.95 | 0.20 | -0.82 | 0.39 | -0.16 | 0.90 | -0.68 | 0.53 |
| SDHD | -0.93 | 0.24 | -0.93 | 0.24 | 0.15 | 0.90 | -0.39 | 0.75 | 0.81 | 0.40 | -0.19 | 0.88 | -0.67 | 0.53 | 0.96 | 0.18 | -0.82 | 0.39 |
| Complex III | | | | | | | | | | | | | | | | | | |
| UQCRB | 0.85 | 0.35 | 0.85 | 0.36 | -0.33 | 0.79 | 0.55 | 0.63 | -0.90 | 0.29 | 0.36 | 0.76 | 0.53 | 0.65 | -0.99 | 0.06 | 0.70 | 0.51 |
| UQCRC1 | 0.23 | 0.85 | 0.23 | 0.85 | 1.00 | 0.00 | -0.97 | 0.16 | 0.70 | 0.50 | -1.00 | 0.03 | 0.64 | 0.56 | 0.41 | 0.73 | 0.45 | 0.70 |
| UQCRC2 | -0.68 | 0.52 | -0.69 | 0.52 | -0.86 | 0.34 | 0.72 | 0.49 | -0.25 | 0.84 | 0.84 | 0.36 | -0.94 | 0.23 | 0.09 | 0.94 | -0.84 | 0.37 |
| UQCRH | 0.78 | 0.43 | 0.78 | 0.43 | 0.79 | 0.42 | -0.61 | 0.58 | 0.12 | 0.92 | -0.76 | 0.45 | 0.98 | 0.14 | -0.23 | 0.85 | 0.90 | 0.28 |
| UQCRQ | 0.90 | 0.29 | 0.90 | 0.29 | 0.63 | 0.57 | -0.42 | 0.73 | -0.11 | 0.93 | -0.59 | 0.60 | 1.00 | 0.00 | -0.45 | 0.71 | 0.98 | 0.13 |
| UQCR10 | 0.50 | 0.66 | 0.51 | 0.66 | 0.95 | 0.19 | -0.85 | 0.35 | 0.46 | 0.69 | -0.94 | 0.22 | 0.83 | 0.37 | 0.13 | 0.92 | 0.69 | 0.51 |
| UQCR11 | -0.29 | 0.81 | -0.30 | 0.81 | -1.00 | 0.05 | 0.95 | 0.20 | -0.65 | 0.55 | 0.99 | 0.07 | -0.69 | 0.52 | -0.35 | 0.77 | -0.51 | 0.66 |
| CYCS | -0.36 | 0.76 | -0.36 | 0.77 | 0.83 | 0.38 | -0.94 | 0.22 | 0.98 | 0.12 | -0.85 | 0.35 | 0.09 | 0.94 | 0.86 | 0.34 | -0.13 | 0.92 |
| Complex IV | | | | | | | | | | | | | | | | | | |
| COX4I1 | 0.98 | 0.14 | 0.98 | 0.13 | 0.42 | 0.72 | -0.19 | 0.88 | -0.34 | 0.78 | -0.39 | 0.75 | 0.97 | 0.16 | -0.64 | 0.55 | 1.00 | 0.02 |
| COX4I2 | 0.13 | 0.92 | 0.14 | 0.91 | 1.00 | 0.06 | -0.99 | 0.10 | 0.77 | 0.44 | -1.00 | 0.03 | 0.56 | 0.62 | 0.50 | 0.67 | 0.36 | 0.76 |
| COX5A | 0.40 | 0.74 | 0.40 | 0.73 | 0.98 | 0.12 | -0.91 | 0.28 | 0.56 | 0.62 | -0.97 | 0.15 | 0.77 | 0.44 | 0.24 | 0.84 | 0.61 | 0.58 |
| COX5B | -0.23 | 0.85 | -0.23 | 0.85 | 0.90 | 0.29 | -0.98 | 0.13 | 0.95 | 0.21 | -0.91 | 0.26 | 0.23 | 0.86 | 0.78 | 0.43 | 0.01 | 0.99 |
| COX6A1 | 0.93 | 0.24 | 0.93 | 0.23 | 0.56 | 0.62 | -0.34 | 0.78 | -0.19 | 0.88 | -0.53 | 0.65 | 1.00 | 0.06 | -0.52 | 0.66 | 0.99 | 0.08 |
| COX6A2 | 0.23 | 0.85 | 0.23 | 0.86 | -0.90 | 0.29 | 0.98 | 0.13 | -0.95 | 0.21 | 0.92 | 0.26 | -0.23 | 0.85 | -0.78 | 0.43 | -0.01 | 0.99 |
| COX6B1 | 1.00 | 0.04 | 1.00 | 0.04 | 0.17 | 0.89 | 0.08 | 0.95 | -0.58 | 0.61 | -0.13 | 0.92 | 0.87 | 0.33 | -0.82 | 0.38 | 0.96 | 0.19 |
| COX6B2 | -0.93 | 0.24 | -0.93 | 0.24 | -0.57 | 0.61 | 0.36 | 0.77 | 0.18 | 0.89 | 0.54 | 0.64 | -1.00 | 0.05 | 0.51 | 0.66 | -0.99 | 0.09 |
| COX6C | 0.86 | 0.34 | 0.86 | 0.34 | 0.69 | 0.51 | -0.50 | 0.67 | -0.02 | 0.99 | -0.66 | 0.54 | 1.00 | 0.05 | -0.37 | 0.76 | 0.96 | 0.19 |
| COX7A1 | -0.52 | 0.65 | -0.52 | 0.65 | 0.71 | 0.49 | -0.86 | 0.34 | 1.00 | 0.01 | -0.74 | 0.47 | -0.09 | 0.94 | 0.93 | 0.23 | -0.31 | 0.80 |
| COX7A2 | 0.96 | 0.19 | 0.95 | 0.19 | -0.08 | 0.95 | 0.32 | 0.80 | -0.76 | 0.45 | 0.12 | 0.93 | 0.73 | 0.48 | -0.94 | 0.23 | 0.86 | 0.34 |
| COX7B | 0.21 | 0.86 | 0.22 | 0.86 | 1.00 | 0.01 | -0.97 | 0.15 | 0.71 | 0.49 | -1.00 | 0.02 | 0.62 | 0.57 | 0.43 | 0.72 | 0.44 | 0.71 |
| COX7C | 0.94 | 0.22 | 0.94 | 0.21 | 0.53 | 0.64 | -0.31 | 0.80 | -0.22 | 0.86 | -0.50 | 0.67 | 0.99 | 0.08 | -0.54 | 0.63 | 1.00 | 0.06 |
| Complex V | | | | | | | | | | | | | | | | | | |
| ATP5C1 | 0.11 | 0.93 | 0.11 | 0.93 | -0.94 | 0.21 | 1.00 | 0.06 | -0.90 | 0.29 | 0.96 | 0.19 | -0.34 | 0.78 | -0.69 | 0.51 | -0.13 | 0.92 |
| ATP5D | 0.00 | 1.00 | 0.00 | 1.00 | 0.97 | 0.14 | -1.00 | 0.01 | 0.85 | 0.36 | -0.98 | 0.12 | 0.44 | 0.71 | 0.61 | 0.58 | 0.24 | 0.85 |
| ATP5E | -0.55 | 0.63 | -0.56 | 0.62 | -0.94 | 0.23 | 0.82 | 0.39 | -0.41 | 0.73 | 0.92 | 0.26 | -0.86 | 0.33 | -0.07 | 0.95 | -0.74 | 0.47 |
| ATP5G3 | 0.76 | 0.45 | 0.76 | 0.45 | 0.80 | 0.41 | -0.64 | 0.56 | 0.15 | 0.91 | -0.78 | 0.43 | 0.97 | 0.16 | -0.20 | 0.87 | 0.89 | 0.30 |
| ATP5J | 0.95 | 0.20 | 0.95 | 0.20 | 0.51 | 0.66 | -0.29 | 0.81 | -0.25 | 0.84 | -0.48 | 0.68 | 0.99 | 0.09 | -0.56 | 0.62 | 1.00 | 0.05 |
| ATP5O | 0.82 | 0.39 | 0.82 | 0.39 | 0.75 | 0.46 | -0.56 | 0.62 | 0.06 | 0.96 | -0.72 | 0.49 | 0.99 | 0.10 | -0.29 | 0.81 | 0.93 | 0.24 |
| ATP5S | -0.84 | 0.36 | -0.84 | 0.36 | -0.71 | 0.50 | 0.52 | 0.65 | -0.01 | 1.00 | 0.68 | 0.52 | -0.99 | 0.07 | 0.34 | 0.78 | -0.95 | 0.21 |

## Slide 12
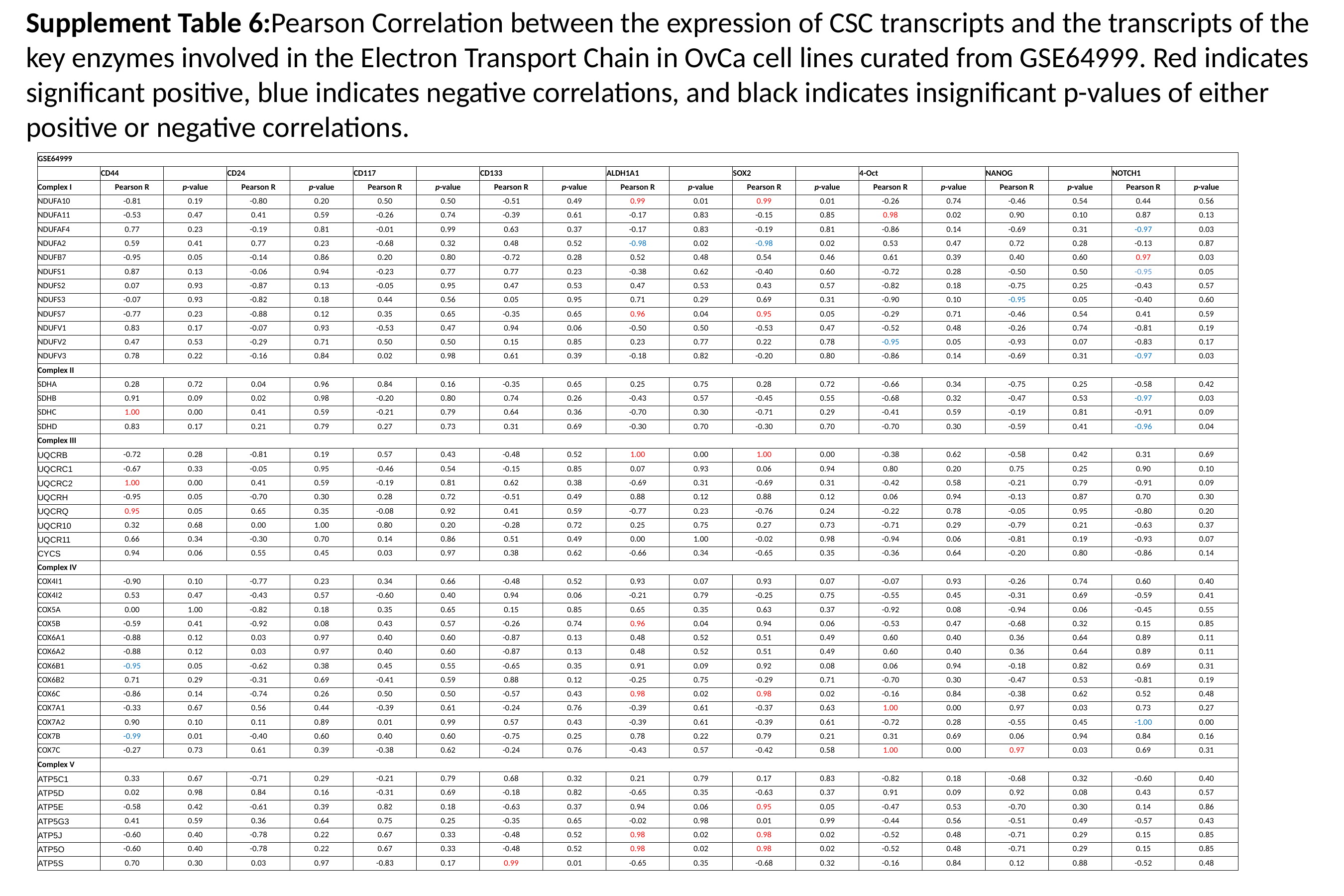

Supplement Table 6:Pearson Correlation between the expression of CSC transcripts and the transcripts of the key enzymes involved in the Electron Transport Chain in OvCa cell lines curated from GSE64999. Red indicates significant positive, blue indicates negative correlations, and black indicates insignificant p-values of either positive or negative correlations.
| GSE64999 | | | | | | | | | | | | | | | | | | |
| --- | --- | --- | --- | --- | --- | --- | --- | --- | --- | --- | --- | --- | --- | --- | --- | --- | --- | --- |
| | CD44 | | CD24 | | CD117 | | CD133 | | ALDH1A1 | | SOX2 | | 4-Oct | | NANOG | | NOTCH1 | |
| Complex I | Pearson R | p-value | Pearson R | p-value | Pearson R | p-value | Pearson R | p-value | Pearson R | p-value | Pearson R | p-value | Pearson R | p-value | Pearson R | p-value | Pearson R | p-value |
| NDUFA10 | -0.81 | 0.19 | -0.80 | 0.20 | 0.50 | 0.50 | -0.51 | 0.49 | 0.99 | 0.01 | 0.99 | 0.01 | -0.26 | 0.74 | -0.46 | 0.54 | 0.44 | 0.56 |
| NDUFA11 | -0.53 | 0.47 | 0.41 | 0.59 | -0.26 | 0.74 | -0.39 | 0.61 | -0.17 | 0.83 | -0.15 | 0.85 | 0.98 | 0.02 | 0.90 | 0.10 | 0.87 | 0.13 |
| NDUFAF4 | 0.77 | 0.23 | -0.19 | 0.81 | -0.01 | 0.99 | 0.63 | 0.37 | -0.17 | 0.83 | -0.19 | 0.81 | -0.86 | 0.14 | -0.69 | 0.31 | -0.97 | 0.03 |
| NDUFA2 | 0.59 | 0.41 | 0.77 | 0.23 | -0.68 | 0.32 | 0.48 | 0.52 | -0.98 | 0.02 | -0.98 | 0.02 | 0.53 | 0.47 | 0.72 | 0.28 | -0.13 | 0.87 |
| NDUFB7 | -0.95 | 0.05 | -0.14 | 0.86 | 0.20 | 0.80 | -0.72 | 0.28 | 0.52 | 0.48 | 0.54 | 0.46 | 0.61 | 0.39 | 0.40 | 0.60 | 0.97 | 0.03 |
| NDUFS1 | 0.87 | 0.13 | -0.06 | 0.94 | -0.23 | 0.77 | 0.77 | 0.23 | -0.38 | 0.62 | -0.40 | 0.60 | -0.72 | 0.28 | -0.50 | 0.50 | -0.95 | 0.05 |
| NDUFS2 | 0.07 | 0.93 | -0.87 | 0.13 | -0.05 | 0.95 | 0.47 | 0.53 | 0.47 | 0.53 | 0.43 | 0.57 | -0.82 | 0.18 | -0.75 | 0.25 | -0.43 | 0.57 |
| NDUFS3 | -0.07 | 0.93 | -0.82 | 0.18 | 0.44 | 0.56 | 0.05 | 0.95 | 0.71 | 0.29 | 0.69 | 0.31 | -0.90 | 0.10 | -0.95 | 0.05 | -0.40 | 0.60 |
| NDUFS7 | -0.77 | 0.23 | -0.88 | 0.12 | 0.35 | 0.65 | -0.35 | 0.65 | 0.96 | 0.04 | 0.95 | 0.05 | -0.29 | 0.71 | -0.46 | 0.54 | 0.41 | 0.59 |
| NDUFV1 | 0.83 | 0.17 | -0.07 | 0.93 | -0.53 | 0.47 | 0.94 | 0.06 | -0.50 | 0.50 | -0.53 | 0.47 | -0.52 | 0.48 | -0.26 | 0.74 | -0.81 | 0.19 |
| NDUFV2 | 0.47 | 0.53 | -0.29 | 0.71 | 0.50 | 0.50 | 0.15 | 0.85 | 0.23 | 0.77 | 0.22 | 0.78 | -0.95 | 0.05 | -0.93 | 0.07 | -0.83 | 0.17 |
| NDUFV3 | 0.78 | 0.22 | -0.16 | 0.84 | 0.02 | 0.98 | 0.61 | 0.39 | -0.18 | 0.82 | -0.20 | 0.80 | -0.86 | 0.14 | -0.69 | 0.31 | -0.97 | 0.03 |
| Complex II | | | | | | | | | | | | | | | | | | |
| SDHA | 0.28 | 0.72 | 0.04 | 0.96 | 0.84 | 0.16 | -0.35 | 0.65 | 0.25 | 0.75 | 0.28 | 0.72 | -0.66 | 0.34 | -0.75 | 0.25 | -0.58 | 0.42 |
| SDHB | 0.91 | 0.09 | 0.02 | 0.98 | -0.20 | 0.80 | 0.74 | 0.26 | -0.43 | 0.57 | -0.45 | 0.55 | -0.68 | 0.32 | -0.47 | 0.53 | -0.97 | 0.03 |
| SDHC | 1.00 | 0.00 | 0.41 | 0.59 | -0.21 | 0.79 | 0.64 | 0.36 | -0.70 | 0.30 | -0.71 | 0.29 | -0.41 | 0.59 | -0.19 | 0.81 | -0.91 | 0.09 |
| SDHD | 0.83 | 0.17 | 0.21 | 0.79 | 0.27 | 0.73 | 0.31 | 0.69 | -0.30 | 0.70 | -0.30 | 0.70 | -0.70 | 0.30 | -0.59 | 0.41 | -0.96 | 0.04 |
| Complex III | | | | | | | | | | | | | | | | | | |
| UQCRB | -0.72 | 0.28 | -0.81 | 0.19 | 0.57 | 0.43 | -0.48 | 0.52 | 1.00 | 0.00 | 1.00 | 0.00 | -0.38 | 0.62 | -0.58 | 0.42 | 0.31 | 0.69 |
| UQCRC1 | -0.67 | 0.33 | -0.05 | 0.95 | -0.46 | 0.54 | -0.15 | 0.85 | 0.07 | 0.93 | 0.06 | 0.94 | 0.80 | 0.20 | 0.75 | 0.25 | 0.90 | 0.10 |
| UQCRC2 | 1.00 | 0.00 | 0.41 | 0.59 | -0.19 | 0.81 | 0.62 | 0.38 | -0.69 | 0.31 | -0.69 | 0.31 | -0.42 | 0.58 | -0.21 | 0.79 | -0.91 | 0.09 |
| UQCRH | -0.95 | 0.05 | -0.70 | 0.30 | 0.28 | 0.72 | -0.51 | 0.49 | 0.88 | 0.12 | 0.88 | 0.12 | 0.06 | 0.94 | -0.13 | 0.87 | 0.70 | 0.30 |
| UQCRQ | 0.95 | 0.05 | 0.65 | 0.35 | -0.08 | 0.92 | 0.41 | 0.59 | -0.77 | 0.23 | -0.76 | 0.24 | -0.22 | 0.78 | -0.05 | 0.95 | -0.80 | 0.20 |
| UQCR10 | 0.32 | 0.68 | 0.00 | 1.00 | 0.80 | 0.20 | -0.28 | 0.72 | 0.25 | 0.75 | 0.27 | 0.73 | -0.71 | 0.29 | -0.79 | 0.21 | -0.63 | 0.37 |
| UQCR11 | 0.66 | 0.34 | -0.30 | 0.70 | 0.14 | 0.86 | 0.51 | 0.49 | 0.00 | 1.00 | -0.02 | 0.98 | -0.94 | 0.06 | -0.81 | 0.19 | -0.93 | 0.07 |
| CYCS | 0.94 | 0.06 | 0.55 | 0.45 | 0.03 | 0.97 | 0.38 | 0.62 | -0.66 | 0.34 | -0.65 | 0.35 | -0.36 | 0.64 | -0.20 | 0.80 | -0.86 | 0.14 |
| Complex IV | | | | | | | | | | | | | | | | | | |
| COX4I1 | -0.90 | 0.10 | -0.77 | 0.23 | 0.34 | 0.66 | -0.48 | 0.52 | 0.93 | 0.07 | 0.93 | 0.07 | -0.07 | 0.93 | -0.26 | 0.74 | 0.60 | 0.40 |
| COX4I2 | 0.53 | 0.47 | -0.43 | 0.57 | -0.60 | 0.40 | 0.94 | 0.06 | -0.21 | 0.79 | -0.25 | 0.75 | -0.55 | 0.45 | -0.31 | 0.69 | -0.59 | 0.41 |
| COX5A | 0.00 | 1.00 | -0.82 | 0.18 | 0.35 | 0.65 | 0.15 | 0.85 | 0.65 | 0.35 | 0.63 | 0.37 | -0.92 | 0.08 | -0.94 | 0.06 | -0.45 | 0.55 |
| COX5B | -0.59 | 0.41 | -0.92 | 0.08 | 0.43 | 0.57 | -0.26 | 0.74 | 0.96 | 0.04 | 0.94 | 0.06 | -0.53 | 0.47 | -0.68 | 0.32 | 0.15 | 0.85 |
| COX6A1 | -0.88 | 0.12 | 0.03 | 0.97 | 0.40 | 0.60 | -0.87 | 0.13 | 0.48 | 0.52 | 0.51 | 0.49 | 0.60 | 0.40 | 0.36 | 0.64 | 0.89 | 0.11 |
| COX6A2 | -0.88 | 0.12 | 0.03 | 0.97 | 0.40 | 0.60 | -0.87 | 0.13 | 0.48 | 0.52 | 0.51 | 0.49 | 0.60 | 0.40 | 0.36 | 0.64 | 0.89 | 0.11 |
| COX6B1 | -0.95 | 0.05 | -0.62 | 0.38 | 0.45 | 0.55 | -0.65 | 0.35 | 0.91 | 0.09 | 0.92 | 0.08 | 0.06 | 0.94 | -0.18 | 0.82 | 0.69 | 0.31 |
| COX6B2 | 0.71 | 0.29 | -0.31 | 0.69 | -0.41 | 0.59 | 0.88 | 0.12 | -0.25 | 0.75 | -0.29 | 0.71 | -0.70 | 0.30 | -0.47 | 0.53 | -0.81 | 0.19 |
| COX6C | -0.86 | 0.14 | -0.74 | 0.26 | 0.50 | 0.50 | -0.57 | 0.43 | 0.98 | 0.02 | 0.98 | 0.02 | -0.16 | 0.84 | -0.38 | 0.62 | 0.52 | 0.48 |
| COX7A1 | -0.33 | 0.67 | 0.56 | 0.44 | -0.39 | 0.61 | -0.24 | 0.76 | -0.39 | 0.61 | -0.37 | 0.63 | 1.00 | 0.00 | 0.97 | 0.03 | 0.73 | 0.27 |
| COX7A2 | 0.90 | 0.10 | 0.11 | 0.89 | 0.01 | 0.99 | 0.57 | 0.43 | -0.39 | 0.61 | -0.39 | 0.61 | -0.72 | 0.28 | -0.55 | 0.45 | -1.00 | 0.00 |
| COX7B | -0.99 | 0.01 | -0.40 | 0.60 | 0.40 | 0.60 | -0.75 | 0.25 | 0.78 | 0.22 | 0.79 | 0.21 | 0.31 | 0.69 | 0.06 | 0.94 | 0.84 | 0.16 |
| COX7C | -0.27 | 0.73 | 0.61 | 0.39 | -0.38 | 0.62 | -0.24 | 0.76 | -0.43 | 0.57 | -0.42 | 0.58 | 1.00 | 0.00 | 0.97 | 0.03 | 0.69 | 0.31 |
| Complex V | | | | | | | | | | | | | | | | | | |
| ATP5C1 | 0.33 | 0.67 | -0.71 | 0.29 | -0.21 | 0.79 | 0.68 | 0.32 | 0.21 | 0.79 | 0.17 | 0.83 | -0.82 | 0.18 | -0.68 | 0.32 | -0.60 | 0.40 |
| ATP5D | 0.02 | 0.98 | 0.84 | 0.16 | -0.31 | 0.69 | -0.18 | 0.82 | -0.65 | 0.35 | -0.63 | 0.37 | 0.91 | 0.09 | 0.92 | 0.08 | 0.43 | 0.57 |
| ATP5E | -0.58 | 0.42 | -0.61 | 0.39 | 0.82 | 0.18 | -0.63 | 0.37 | 0.94 | 0.06 | 0.95 | 0.05 | -0.47 | 0.53 | -0.70 | 0.30 | 0.14 | 0.86 |
| ATP5G3 | 0.41 | 0.59 | 0.36 | 0.64 | 0.75 | 0.25 | -0.35 | 0.65 | -0.02 | 0.98 | 0.01 | 0.99 | -0.44 | 0.56 | -0.51 | 0.49 | -0.57 | 0.43 |
| ATP5J | -0.60 | 0.40 | -0.78 | 0.22 | 0.67 | 0.33 | -0.48 | 0.52 | 0.98 | 0.02 | 0.98 | 0.02 | -0.52 | 0.48 | -0.71 | 0.29 | 0.15 | 0.85 |
| ATP5O | -0.60 | 0.40 | -0.78 | 0.22 | 0.67 | 0.33 | -0.48 | 0.52 | 0.98 | 0.02 | 0.98 | 0.02 | -0.52 | 0.48 | -0.71 | 0.29 | 0.15 | 0.85 |
| ATP5S | 0.70 | 0.30 | 0.03 | 0.97 | -0.83 | 0.17 | 0.99 | 0.01 | -0.65 | 0.35 | -0.68 | 0.32 | -0.16 | 0.84 | 0.12 | 0.88 | -0.52 | 0.48 |

## Slide 13
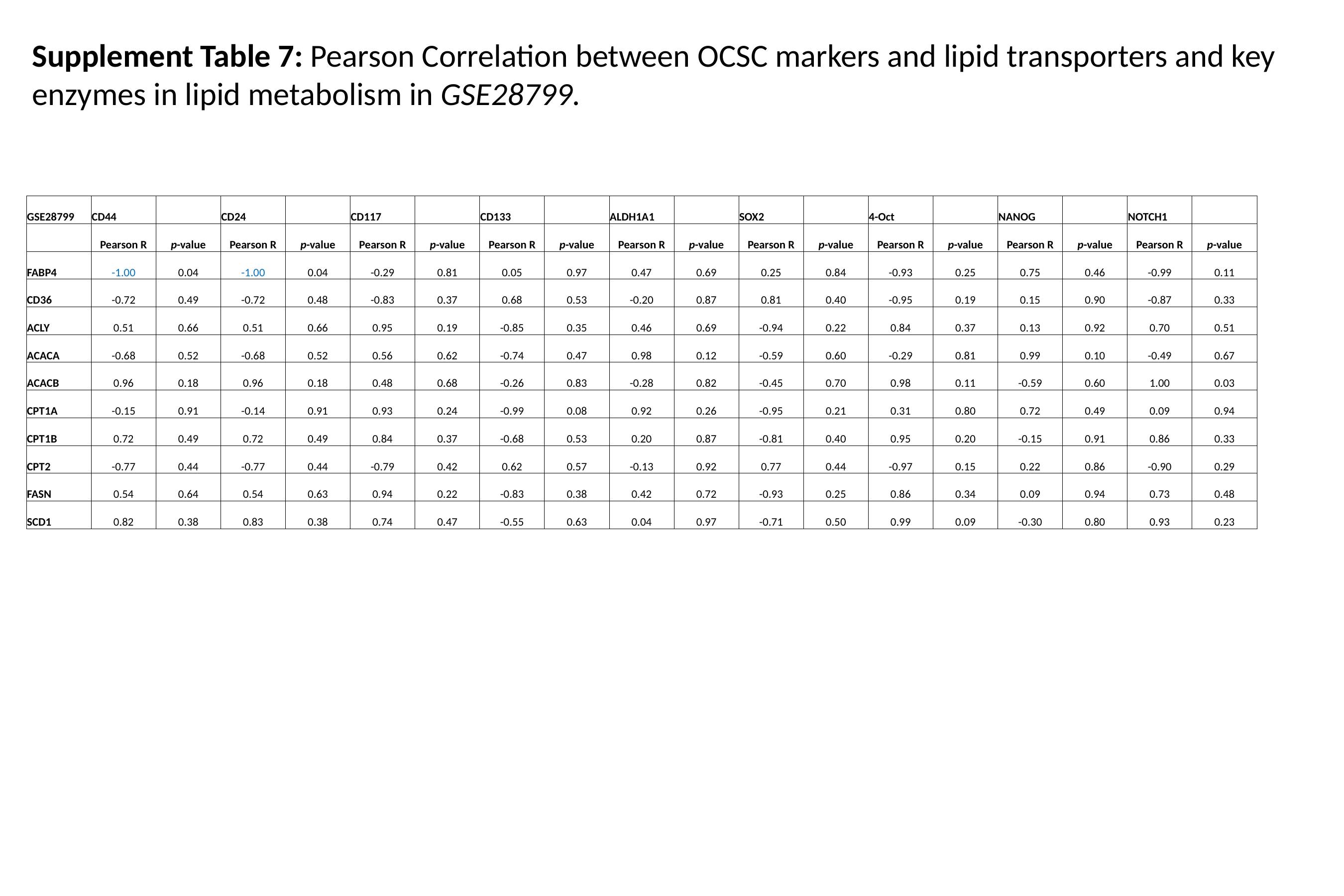

Supplement Table 7: Pearson Correlation between OCSC markers and lipid transporters and key enzymes in lipid metabolism in GSE28799.
| GSE28799 | CD44 | | CD24 | | CD117 | | CD133 | | ALDH1A1 | | SOX2 | | 4-Oct | | NANOG | | NOTCH1 | |
| --- | --- | --- | --- | --- | --- | --- | --- | --- | --- | --- | --- | --- | --- | --- | --- | --- | --- | --- |
| | Pearson R | p-value | Pearson R | p-value | Pearson R | p-value | Pearson R | p-value | Pearson R | p-value | Pearson R | p-value | Pearson R | p-value | Pearson R | p-value | Pearson R | p-value |
| FABP4 | -1.00 | 0.04 | -1.00 | 0.04 | -0.29 | 0.81 | 0.05 | 0.97 | 0.47 | 0.69 | 0.25 | 0.84 | -0.93 | 0.25 | 0.75 | 0.46 | -0.99 | 0.11 |
| CD36 | -0.72 | 0.49 | -0.72 | 0.48 | -0.83 | 0.37 | 0.68 | 0.53 | -0.20 | 0.87 | 0.81 | 0.40 | -0.95 | 0.19 | 0.15 | 0.90 | -0.87 | 0.33 |
| ACLY | 0.51 | 0.66 | 0.51 | 0.66 | 0.95 | 0.19 | -0.85 | 0.35 | 0.46 | 0.69 | -0.94 | 0.22 | 0.84 | 0.37 | 0.13 | 0.92 | 0.70 | 0.51 |
| ACACA | -0.68 | 0.52 | -0.68 | 0.52 | 0.56 | 0.62 | -0.74 | 0.47 | 0.98 | 0.12 | -0.59 | 0.60 | -0.29 | 0.81 | 0.99 | 0.10 | -0.49 | 0.67 |
| ACACB | 0.96 | 0.18 | 0.96 | 0.18 | 0.48 | 0.68 | -0.26 | 0.83 | -0.28 | 0.82 | -0.45 | 0.70 | 0.98 | 0.11 | -0.59 | 0.60 | 1.00 | 0.03 |
| CPT1A | -0.15 | 0.91 | -0.14 | 0.91 | 0.93 | 0.24 | -0.99 | 0.08 | 0.92 | 0.26 | -0.95 | 0.21 | 0.31 | 0.80 | 0.72 | 0.49 | 0.09 | 0.94 |
| CPT1B | 0.72 | 0.49 | 0.72 | 0.49 | 0.84 | 0.37 | -0.68 | 0.53 | 0.20 | 0.87 | -0.81 | 0.40 | 0.95 | 0.20 | -0.15 | 0.91 | 0.86 | 0.33 |
| CPT2 | -0.77 | 0.44 | -0.77 | 0.44 | -0.79 | 0.42 | 0.62 | 0.57 | -0.13 | 0.92 | 0.77 | 0.44 | -0.97 | 0.15 | 0.22 | 0.86 | -0.90 | 0.29 |
| FASN | 0.54 | 0.64 | 0.54 | 0.63 | 0.94 | 0.22 | -0.83 | 0.38 | 0.42 | 0.72 | -0.93 | 0.25 | 0.86 | 0.34 | 0.09 | 0.94 | 0.73 | 0.48 |
| SCD1 | 0.82 | 0.38 | 0.83 | 0.38 | 0.74 | 0.47 | -0.55 | 0.63 | 0.04 | 0.97 | -0.71 | 0.50 | 0.99 | 0.09 | -0.30 | 0.80 | 0.93 | 0.23 |

## Slide 14
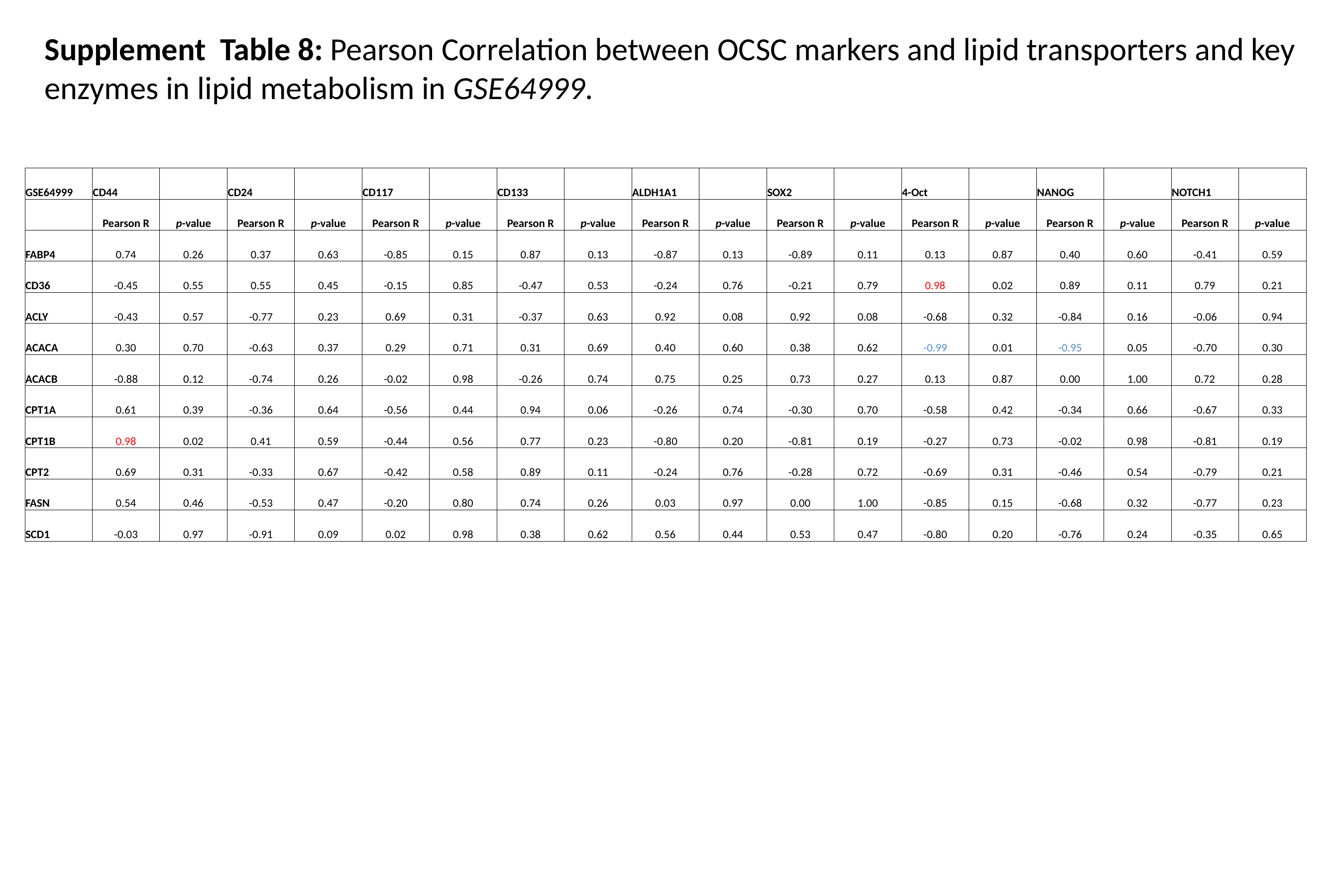

Supplement Table 8: Pearson Correlation between OCSC markers and lipid transporters and key enzymes in lipid metabolism in GSE64999.
| GSE64999 | CD44 | | CD24 | | CD117 | | CD133 | | ALDH1A1 | | SOX2 | | 4-Oct | | NANOG | | NOTCH1 | |
| --- | --- | --- | --- | --- | --- | --- | --- | --- | --- | --- | --- | --- | --- | --- | --- | --- | --- | --- |
| | Pearson R | p-value | Pearson R | p-value | Pearson R | p-value | Pearson R | p-value | Pearson R | p-value | Pearson R | p-value | Pearson R | p-value | Pearson R | p-value | Pearson R | p-value |
| FABP4 | 0.74 | 0.26 | 0.37 | 0.63 | -0.85 | 0.15 | 0.87 | 0.13 | -0.87 | 0.13 | -0.89 | 0.11 | 0.13 | 0.87 | 0.40 | 0.60 | -0.41 | 0.59 |
| CD36 | -0.45 | 0.55 | 0.55 | 0.45 | -0.15 | 0.85 | -0.47 | 0.53 | -0.24 | 0.76 | -0.21 | 0.79 | 0.98 | 0.02 | 0.89 | 0.11 | 0.79 | 0.21 |
| ACLY | -0.43 | 0.57 | -0.77 | 0.23 | 0.69 | 0.31 | -0.37 | 0.63 | 0.92 | 0.08 | 0.92 | 0.08 | -0.68 | 0.32 | -0.84 | 0.16 | -0.06 | 0.94 |
| ACACA | 0.30 | 0.70 | -0.63 | 0.37 | 0.29 | 0.71 | 0.31 | 0.69 | 0.40 | 0.60 | 0.38 | 0.62 | -0.99 | 0.01 | -0.95 | 0.05 | -0.70 | 0.30 |
| ACACB | -0.88 | 0.12 | -0.74 | 0.26 | -0.02 | 0.98 | -0.26 | 0.74 | 0.75 | 0.25 | 0.73 | 0.27 | 0.13 | 0.87 | 0.00 | 1.00 | 0.72 | 0.28 |
| CPT1A | 0.61 | 0.39 | -0.36 | 0.64 | -0.56 | 0.44 | 0.94 | 0.06 | -0.26 | 0.74 | -0.30 | 0.70 | -0.58 | 0.42 | -0.34 | 0.66 | -0.67 | 0.33 |
| CPT1B | 0.98 | 0.02 | 0.41 | 0.59 | -0.44 | 0.56 | 0.77 | 0.23 | -0.80 | 0.20 | -0.81 | 0.19 | -0.27 | 0.73 | -0.02 | 0.98 | -0.81 | 0.19 |
| CPT2 | 0.69 | 0.31 | -0.33 | 0.67 | -0.42 | 0.58 | 0.89 | 0.11 | -0.24 | 0.76 | -0.28 | 0.72 | -0.69 | 0.31 | -0.46 | 0.54 | -0.79 | 0.21 |
| FASN | 0.54 | 0.46 | -0.53 | 0.47 | -0.20 | 0.80 | 0.74 | 0.26 | 0.03 | 0.97 | 0.00 | 1.00 | -0.85 | 0.15 | -0.68 | 0.32 | -0.77 | 0.23 |
| SCD1 | -0.03 | 0.97 | -0.91 | 0.09 | 0.02 | 0.98 | 0.38 | 0.62 | 0.56 | 0.44 | 0.53 | 0.47 | -0.80 | 0.20 | -0.76 | 0.24 | -0.35 | 0.65 |

## Slide 15
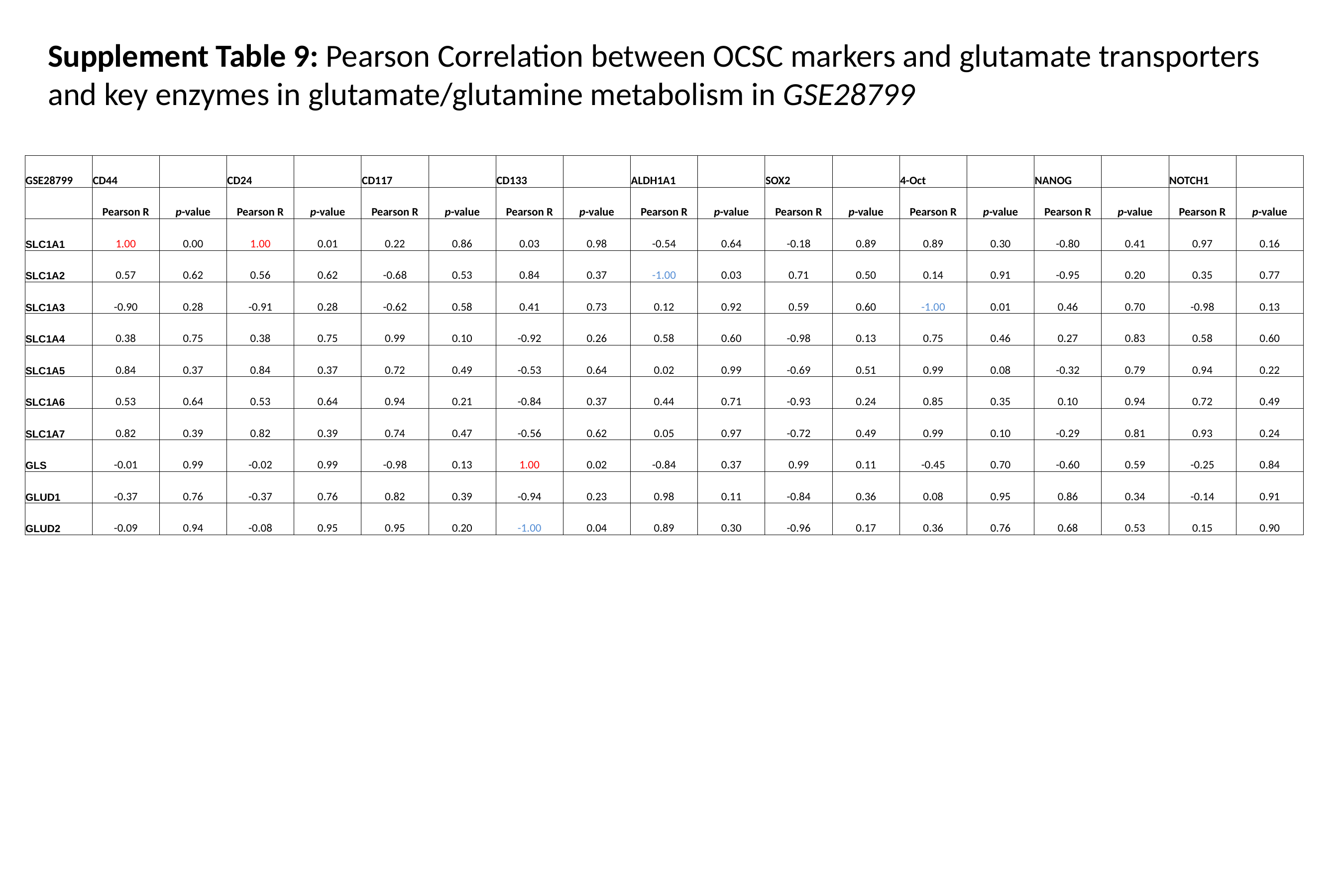

Supplement Table 9: Pearson Correlation between OCSC markers and glutamate transporters and key enzymes in glutamate/glutamine metabolism in GSE28799
| GSE28799 | CD44 | | CD24 | | CD117 | | CD133 | | ALDH1A1 | | SOX2 | | 4-Oct | | NANOG | | NOTCH1 | |
| --- | --- | --- | --- | --- | --- | --- | --- | --- | --- | --- | --- | --- | --- | --- | --- | --- | --- | --- |
| | Pearson R | p-value | Pearson R | p-value | Pearson R | p-value | Pearson R | p-value | Pearson R | p-value | Pearson R | p-value | Pearson R | p-value | Pearson R | p-value | Pearson R | p-value |
| SLC1A1 | 1.00 | 0.00 | 1.00 | 0.01 | 0.22 | 0.86 | 0.03 | 0.98 | -0.54 | 0.64 | -0.18 | 0.89 | 0.89 | 0.30 | -0.80 | 0.41 | 0.97 | 0.16 |
| SLC1A2 | 0.57 | 0.62 | 0.56 | 0.62 | -0.68 | 0.53 | 0.84 | 0.37 | -1.00 | 0.03 | 0.71 | 0.50 | 0.14 | 0.91 | -0.95 | 0.20 | 0.35 | 0.77 |
| SLC1A3 | -0.90 | 0.28 | -0.91 | 0.28 | -0.62 | 0.58 | 0.41 | 0.73 | 0.12 | 0.92 | 0.59 | 0.60 | -1.00 | 0.01 | 0.46 | 0.70 | -0.98 | 0.13 |
| SLC1A4 | 0.38 | 0.75 | 0.38 | 0.75 | 0.99 | 0.10 | -0.92 | 0.26 | 0.58 | 0.60 | -0.98 | 0.13 | 0.75 | 0.46 | 0.27 | 0.83 | 0.58 | 0.60 |
| SLC1A5 | 0.84 | 0.37 | 0.84 | 0.37 | 0.72 | 0.49 | -0.53 | 0.64 | 0.02 | 0.99 | -0.69 | 0.51 | 0.99 | 0.08 | -0.32 | 0.79 | 0.94 | 0.22 |
| SLC1A6 | 0.53 | 0.64 | 0.53 | 0.64 | 0.94 | 0.21 | -0.84 | 0.37 | 0.44 | 0.71 | -0.93 | 0.24 | 0.85 | 0.35 | 0.10 | 0.94 | 0.72 | 0.49 |
| SLC1A7 | 0.82 | 0.39 | 0.82 | 0.39 | 0.74 | 0.47 | -0.56 | 0.62 | 0.05 | 0.97 | -0.72 | 0.49 | 0.99 | 0.10 | -0.29 | 0.81 | 0.93 | 0.24 |
| GLS | -0.01 | 0.99 | -0.02 | 0.99 | -0.98 | 0.13 | 1.00 | 0.02 | -0.84 | 0.37 | 0.99 | 0.11 | -0.45 | 0.70 | -0.60 | 0.59 | -0.25 | 0.84 |
| GLUD1 | -0.37 | 0.76 | -0.37 | 0.76 | 0.82 | 0.39 | -0.94 | 0.23 | 0.98 | 0.11 | -0.84 | 0.36 | 0.08 | 0.95 | 0.86 | 0.34 | -0.14 | 0.91 |
| GLUD2 | -0.09 | 0.94 | -0.08 | 0.95 | 0.95 | 0.20 | -1.00 | 0.04 | 0.89 | 0.30 | -0.96 | 0.17 | 0.36 | 0.76 | 0.68 | 0.53 | 0.15 | 0.90 |

## Slide 16
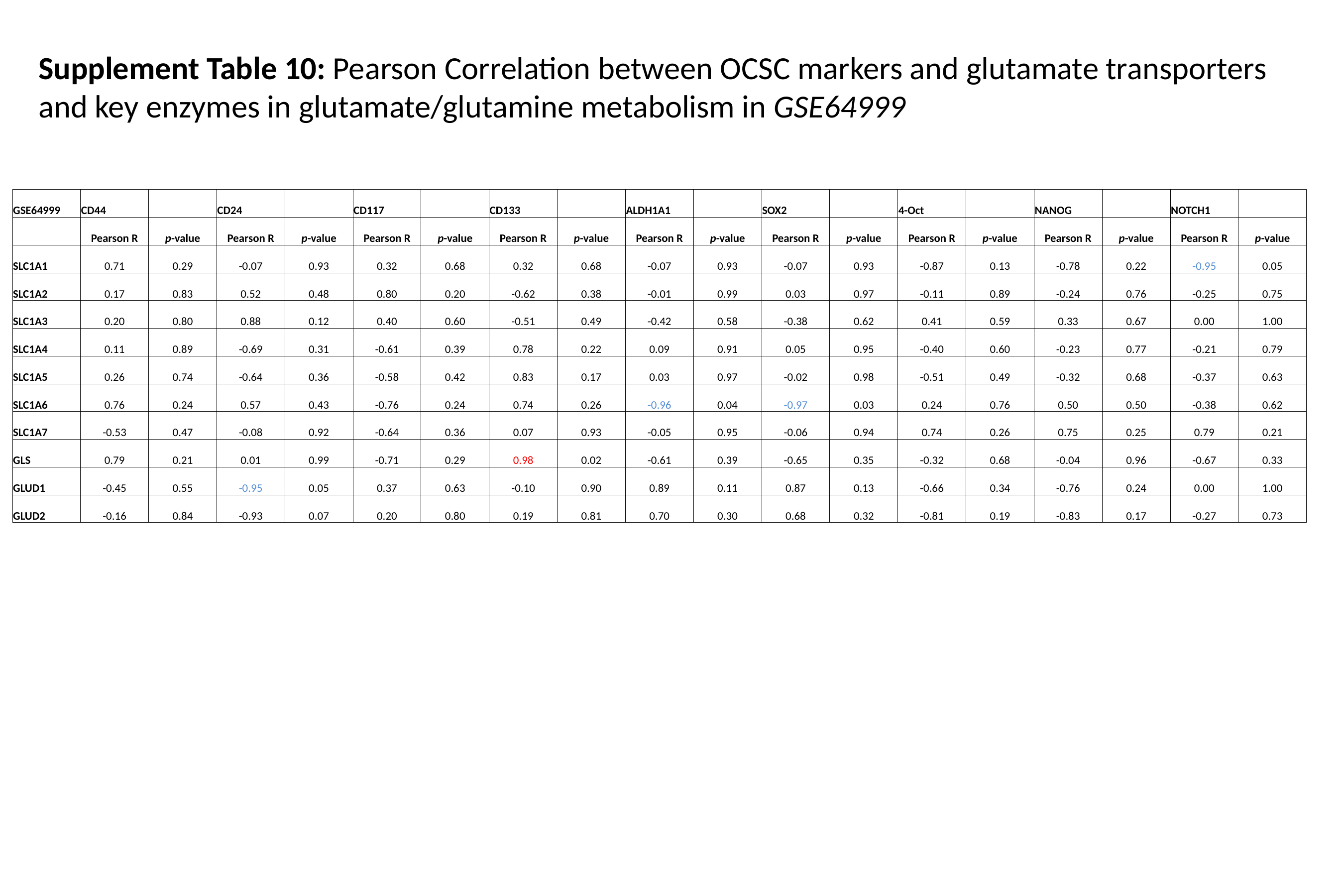

Supplement Table 10: Pearson Correlation between OCSC markers and glutamate transporters and key enzymes in glutamate/glutamine metabolism in GSE64999
| GSE64999 | CD44 | | CD24 | | CD117 | | CD133 | | ALDH1A1 | | SOX2 | | 4-Oct | | NANOG | | NOTCH1 | |
| --- | --- | --- | --- | --- | --- | --- | --- | --- | --- | --- | --- | --- | --- | --- | --- | --- | --- | --- |
| | Pearson R | p-value | Pearson R | p-value | Pearson R | p-value | Pearson R | p-value | Pearson R | p-value | Pearson R | p-value | Pearson R | p-value | Pearson R | p-value | Pearson R | p-value |
| SLC1A1 | 0.71 | 0.29 | -0.07 | 0.93 | 0.32 | 0.68 | 0.32 | 0.68 | -0.07 | 0.93 | -0.07 | 0.93 | -0.87 | 0.13 | -0.78 | 0.22 | -0.95 | 0.05 |
| SLC1A2 | 0.17 | 0.83 | 0.52 | 0.48 | 0.80 | 0.20 | -0.62 | 0.38 | -0.01 | 0.99 | 0.03 | 0.97 | -0.11 | 0.89 | -0.24 | 0.76 | -0.25 | 0.75 |
| SLC1A3 | 0.20 | 0.80 | 0.88 | 0.12 | 0.40 | 0.60 | -0.51 | 0.49 | -0.42 | 0.58 | -0.38 | 0.62 | 0.41 | 0.59 | 0.33 | 0.67 | 0.00 | 1.00 |
| SLC1A4 | 0.11 | 0.89 | -0.69 | 0.31 | -0.61 | 0.39 | 0.78 | 0.22 | 0.09 | 0.91 | 0.05 | 0.95 | -0.40 | 0.60 | -0.23 | 0.77 | -0.21 | 0.79 |
| SLC1A5 | 0.26 | 0.74 | -0.64 | 0.36 | -0.58 | 0.42 | 0.83 | 0.17 | 0.03 | 0.97 | -0.02 | 0.98 | -0.51 | 0.49 | -0.32 | 0.68 | -0.37 | 0.63 |
| SLC1A6 | 0.76 | 0.24 | 0.57 | 0.43 | -0.76 | 0.24 | 0.74 | 0.26 | -0.96 | 0.04 | -0.97 | 0.03 | 0.24 | 0.76 | 0.50 | 0.50 | -0.38 | 0.62 |
| SLC1A7 | -0.53 | 0.47 | -0.08 | 0.92 | -0.64 | 0.36 | 0.07 | 0.93 | -0.05 | 0.95 | -0.06 | 0.94 | 0.74 | 0.26 | 0.75 | 0.25 | 0.79 | 0.21 |
| GLS | 0.79 | 0.21 | 0.01 | 0.99 | -0.71 | 0.29 | 0.98 | 0.02 | -0.61 | 0.39 | -0.65 | 0.35 | -0.32 | 0.68 | -0.04 | 0.96 | -0.67 | 0.33 |
| GLUD1 | -0.45 | 0.55 | -0.95 | 0.05 | 0.37 | 0.63 | -0.10 | 0.90 | 0.89 | 0.11 | 0.87 | 0.13 | -0.66 | 0.34 | -0.76 | 0.24 | 0.00 | 1.00 |
| GLUD2 | -0.16 | 0.84 | -0.93 | 0.07 | 0.20 | 0.80 | 0.19 | 0.81 | 0.70 | 0.30 | 0.68 | 0.32 | -0.81 | 0.19 | -0.83 | 0.17 | -0.27 | 0.73 |
